# A Machine Vision-Enhanced Framework for Tracking Inclusion Evolution and Enabling Intelligent Cleanliness Control in Industrial-Scale HSLA Steels

**DOI:** 10.3390/ma19010158

**Published:** 2026-01-02

**Authors:** Yong Lyu, Yunhai Jia, Lixia Yang, Weihao Wan, Danyang Zhi, Xuehua Wang, Peifeng Cheng, Haizhou Wang

**Affiliations:** 1 School of Metallurgical and Ecological Engineering, University of Science and Technology Beijing, Beijing 100083, China; 15332728942@163.com; 2 Central Iron & Steel Research Institute, Beijing 100081, China; yanglixia@ncschina.com (L.Y.); wanweihao@ncschina.com (W.W.); zhidanyang@ncschina.com (D.Z.); wangxuehua@ncschina.com (X.W.); chengpeifeng@ncschina.com (P.C.); wanghaizhou@ncschina.com (H.W.)

**Keywords:** High-Strength Low-Alloy (HSLA) steel, non-metallic inclusions, full-range analysis system, inclusion evolution, cleanliness control, intelligent inspection

## Abstract

The quantity, size, and distribution of non-metallic inclusions in High-Strength Low-Alloy (HSLA) steel critically influence its service performance. Conventional detection methods often fail to adequately characterize extreme inclusion distributions in large-section components. This study developed an integrated full-process inclusion analysis system combining high-precision motion control, parallel optical imaging, and laser spectral analysis technologies to achieve rapid and automated identification and compositional analysis of inclusions in meter-scale samples. Through systematic investigation across the industrial process chain—from a dia. 740 mm consumable electrode to a dia. 810 mm electroslag remelting (ESR) ingot and finally to a dia. 400 mm forged billet—key process-specific insights were obtained. The results revealed the effective removal of Type D (globular oxides) inclusions during ESR, with their counts reducing from over 8000 in the electrode to approximately 4000–7000 in the ingot. Concurrently, the mechanism underlying the pronounced enrichment of Type C (silicates) in the ingot tail was elucidated, showing a nearly fourfold increase to 1767 compared to the ingot head, attributed to terminal solidification segregation and flotation dynamics. Subsequent forging further demonstrated exceptional refinement and dispersion of all inclusion types. The billet tail achieved exceptionally high purity, with counts of all inclusion types dropping to extremely low levels (e.g., Types A, B, and C were nearly eliminated), representing a reduction of approximately one order of magnitude. Based on these findings, enhanced process strategies were proposed, including shallow molten pool control, slag system optimization, and multi-dimensional quality monitoring. An intelligent analysis framework integrating a YOLOv11 detection model with spectral feedback was also established. This work provides crucial process knowledge and technological support for achieving the quality control objective of “known and controllable defects” in HSLA steel.

## 1. Introduction

High-Strength Low-Alloy (HSLA) steel, a cornerstone structural material in modern industry, often exhibits significant deviations between its actual service performance and theoretically predicted properties [1,2]. This discrepancy can be fundamentally traced to non-metallic inclusions, which are inherently difficult to eliminate entirely during the steelmaking process [3]. As intrinsic defects, these inclusions systematically compromise the in-service performance of HSLA steel and serve as a critical factor causing its measured mechanical behavior to deviate from the ideal state [4,5,6]. Acting as heterogeneous particles, inclusions severely disrupt the continuity of the steel matrix, creating localized stress concentration sites. They exert a decisive influence on the material’s fatigue performance—under cyclic loading, brittle oxides (e.g., Al_2_O_3_) and non-deformable inclusions readily act as nucleation sites for microcracks, significantly reducing the fatigue limit and service life of components and exhibiting typical “weakest-link” characteristics [7]. Furthermore, inclusions markedly degrade the material’s toughness, leading to a sharp drop in impact energy (particularly transverse impact toughness) and promoting crack propagation, thereby weakening fracture resistance [8]. In terms of macroscopic mechanical properties, inclusions not only impair ductility and plasticity but, more importantly, cause significant mechanical anisotropy due to the directional elongation of plastic inclusions (e.g., MnS) during hot working [9,10,11]. Additionally, in demanding service environments, inclusions act as hydrogen traps, substantially increasing the material’s susceptibility to hydrogen-induced cracking and sulfide stress corrosion cracking [12].

While HSLA steels have achieved significant strength improvements through microalloying design and Thermo-Mechanical Controlled Processing (TMCP), the inclusion control capability of conventional smelting processes remains inadequate for the increasingly stringent service reliability demands in high-end applications such as aerospace fasteners, high-cycle fatigue engineering components, and deep-sea pipeline systems [13]. Numerous failure analysis cases have confirmed that fatigue cracks in automotive chassis components and premature spalling in wind turbine bearings often originate directly from microscopic defects, such as oxide clusters, sulfide banding segregation, or hard, non-deformable inclusions. These defects exhibit significant spatial randomness and statistical concealment within large-scale components. Consequently, traditional quality assessment methods based on small-sized specimens are often inadequate for accurately characterizing the overall inclusion distribution in the entire component, thereby introducing considerable uncertainties into performance prediction and safety evaluation.

Despite significant advancements in advanced metallurgical processes such as LF-VD secondary refining and calcium treatment for the modification of inclusions in HSLA steels, several technical bottlenecks persist in the manufacturing of large-section, ultra-high-strength components [14,15,16]. Current mainstream inclusion modification techniques, such as calcium treatment, magnesium treatment, and rare earth treatment, each offer distinct advantages but are constrained by inherent limitations. Calcium treatment can transform high-melting-point solid Al_2_O_3_ into low-melting-point liquid calcium aluminates (e.g., CaO·Al_2_O_3_), significantly improving steel castability and reducing nozzle clogging. However, its modification efficiency is strongly influenced by the oxygen and sulfur activities in the molten steel, and the treatment effectiveness is often unstable [17]. Magnesium treatment can form thermally stable MgAl_2_O_4_ spinel inclusions, which contribute to improved steel toughness. Nevertheless, magnesium has a low boiling point (1107 °C), leading to significant vaporization losses in the steel melt, posing challenges in process control. Excessive addition can also result in harmful coarse MgO inclusions. Rare earth treatment (e.g., using Ce, La) exhibits a strong affinity for oxygen and sulfur, forming fine, spherical, and thermodynamically stable rare earth oxysulfides (e.g., Ce_2_O_2_S). This effectively spheroidizes MnS inclusions, enhancing steel fatigue life (by 30–50%) and improving isotropic properties. However, rare earth elements are costly and prone to agglomerate into large clusters (reaching 50–100 μm) in the melt. They also readily react with refractories, causing severe nozzle clogging, which limits their widespread industrial application. Critically, these single modifiers often struggle to achieve comprehensive and stable inclusion control in HSLA steels with complex compositions (e.g., high oxygen, high sulfur, or containing microalloying elements like Ti and Nb). Furthermore, the limited sampling area of current international standards (e.g., ASTM E45) fails to capture the extreme sizes and most severe distributions of inclusions in large components. This leads to a systematic underestimation in laboratory assessments, increasing design risks and the potential for in-service failure. These fundamental limitations not only threaten the service safety of critical components but also severely constrain the iterative efficiency of new steel grade development and process optimization.

To achieve breakthroughs in HSLA steels for ultra-high stress, ultra-long life, and extreme environment applications, the quality control philosophy must undergo a fundamental paradigm shift—from merely ‘meeting smelting specifications’ to achieving ‘defects that are known and controllable’. In recent years, ‘synergistic modification’ strategies that combine calcium, magnesium, and rare earth elements have attracted widespread attention due to their potential to leverage the strengths of individual elements while mitigating their drawbacks, representing a promising direction for advanced inclusion engineering. However, the successful implementation of both single and synergistic modification strategies relies on the precise and efficient characterization of inclusion evolution throughout the entire process chain. To address the technical challenge of statistically characterizing inclusions in large-scale metallic materials, this study developed a self-contained, full-range inclusion analysis system. This system integrates cutting-edge technologies, including high-precision motion control, a parallel optical imaging array, micro-area laser spectroscopy, and GPU-accelerated big data processing. It enables rapid, fully automated, full-field identification, localization, and compositional analysis of inclusions in meter-scale samples. This system provides an advanced analytical platform for the development and quality control of high-grade steels, aiming to deliver crucial technological support for achieving the intelligent quality control objective of ‘known and controllable defects’, effectively filling a critical technological gap in this field.

## 2. Materials and Methods

### 2.1. Instrumentation

**Figure 1:**
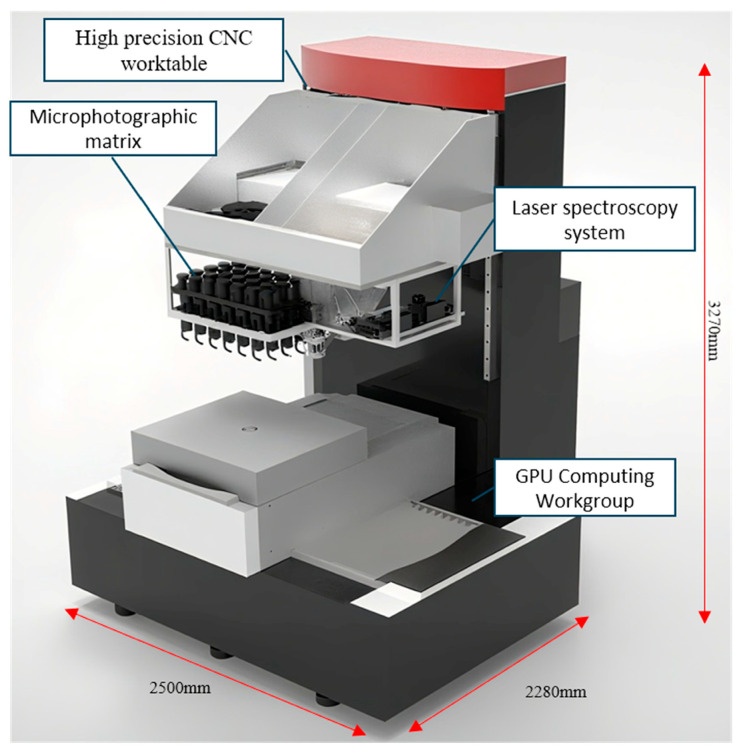
Schematic diagram of the instrument.

By integrating parallel image acquisition with high-performance computing, the metallographic microscope matrix system achieves efficient, full-range analysis of large samples. The system comprises 24 synchronously operating microscopic imaging units. Under the coordination of central control software, these units perform rapid concurrent image acquisition from different regions of the sample surface, significantly enhancing inspection efficiency. Following acquisition, the process seamlessly proceeds to an integrated data processing pipeline. Leveraging the parallel computing power of a GPU, the system performs high-speed preprocessing and intelligent analysis of the massive image dataset. Each metallographic image undergoes a series of processing operations—including cropping, binarization, rotation, scaling, and secondary cropping—within 0.7 s. This ensures that all images maintain consistency in orientation and magnification with the coordinate system of the precision motion stage. Following the automated panoramic stitching of images based on their precise spatial coordinates—which generates a complete metallographic map of the large-scale sample for the rapid identification, localization, and statistics of inclusions—the system further enhances analytical accuracy by integrating with Laser-Induced Breakdown Spectroscopy (LIBS). This coupled setup enables in situ micro-area compositional analysis of typical inclusions previously identified by the microscope matrix, facilitating their qualitative to semi-quantitative determination and thereby delivering reliable data for material performance evaluation and process optimization. Figure 1 shows a schematic diagram of the instrument structure. The detailed instrumentation and operating parameters are provided in Part I of the Supplementary Materials.

### 2.2. Intelligent Detection of Inclusions Using YOLOv11

#### 2.2.1. Data Acquisition and Preprocessing

To achieve accurate inclusion identification and detection, a batch of high-quality metallographic images was systematically acquired. The data were sourced from two complementary approaches: (1) Scanning Electron Microscopy (SEM) was used to image small-sized metallographic samples (individual area ≈ 200 mm^2^), with Energy Dispersive Spectroscopy (EDS) providing reference data on inclusion class, morphology, and location (FESEM, FEI-Quanta FEG 250, USA); (2) Corresponding regions were captured optically using the large-scale microscopic matrix analysis system. Based on SEM-EDS results, all visible inclusions in the optical images were annotated with category and location labels, resulting in approximately 1000 valid annotations. To enhance model generalizability and mitigate data scarcity, a series of data augmentation techniques was applied to the labeled images, including geometric transformations (rotation, flipping) and photometric adjustments (brightness, contrast, noise injection). Finally, the dataset was randomly split into training, validation, and test sets in an 8:1:1 ratio, establishing a robust foundation for model training and evaluation.

The initial annotated dataset comprised approximately 1000 valid inclusions. While this scale is sufficient for initial model development and proof-of-concept, we recognize its potential limitations for capturing the full morphological spectrum and extreme cases of inclusions that may occur under varying industrial conditions. To mitigate the risk of overfitting and enhance the model’s generalizability, a multi-faceted strategy was implemented: (1) Aggressive Data Augmentation: Extensive geometric (rotation, flipping, scaling) and photometric (brightness, contrast, noise) transformations were applied to simulate real-world imaging variances, effectively expanding the dataset’s diversity. (2) Transfer Learning: The model was initialized with weights pre-trained on the large-scale COCO dataset, allowing it to leverage generalized feature extraction capabilities before fine-tuning on our specialized metallographic data. (3) Incremental Learning Loop: The established “detection-verification-optimization” pipeline continuously incorporates newly verified samples (via LIBS or expert review) into the training set, enabling the model to evolve and adapt with ongoing use. These measures collectively ensured that the model learned robust, invariant features of inclusions rather than memorizing the limited training set, as evidenced by its consistent performance across multiple independent samples from different process stages (see Results, Section 3).

#### 2.2.2. YOLOv11 Model Architecture and Technical Advantages

This study employs YOLOv11 as the core detection architecture, which represents the latest single-stage detection framework released by Ultralytics in 2024 and achieves several breakthroughs in precision-speed balance and structural efficiency. By incorporating a C3K2 dynamic convolution module and a C2PSA attention fusion mechanism, YOLOv11 significantly enhances multi-scale feature extraction capability. While preserving high-resolution semantic information, the model reduces parameter count by 22% and improves computational efficiency by 18% compared to YOLOv8. Furthermore, the adoption of a depth-wise separable detection head and an EIoU loss function increases inference speed by 23% and improves bounding box regression accuracy by 2.1%. YOLOv11 demonstrates three key advantages in this application:

End-to-End Training Mechanism: The model reframes object detection as a unified regression problem, performing both target localization and classification in a single forward pass. This streamlines the traditional multi-stage detection pipeline by approximately 40%, significantly improving model iteration speed and cross-material adaptability.

Industrial-Grade Inference Performance: When deployed on an NVIDIA T4 GPU, the system achieves detection speeds exceeding 140 FPS. With TensorRT acceleration, inference latency is reduced to below 7 ms, fully meeting the demands of high-speed online inspection. Meanwhile, it maintains a recall rate above 92% for micron-scale inclusions as small as 15 × 15 pixels.

Open Ecosystem and Extensibility: Support for industry-standard formats such as ONNX and CoreML facilitates deployment on embedded devices and edge computing platforms. Combined with ongoing model optimization techniques, a lightweight version under 3 MB can be realized, ensuring compatibility with complex industrial environments.

#### 2.2.3. Model Training and Optimization Strategy

The model was trained using a combined strategy of transfer learning and incremental optimization. Initialized with YOLOv11 pre-trained weights, input images were uniformly resized to 640 × 640 pixels, and the initial learning rate was set to 1 × 10^−2^. Through 100 epochs of training, the optimal model was selected based on validation performance.

To address the diversity of inclusion morphologies and sample imbalance, a multi-level optimization mechanism was designed: Dynamic label assignment and adaptive anchor optimization strategies were introduced to enhance model sensitivity to small inclusions in complex backgrounds; An incremental learning approach was implemented, where laser system verification results and expert-validated samples were fed back into the training set to enable continuous evolution of detection capability; An SEM-informed attention mechanism was incorporated to improve morphological discrimination of typical inclusions (Types A, B, C, and D). As can be seen from Table 1, the comparative data between YOLOv11 and YOLOv8 (such as differences in mAP, speed, and parameter count) are reasonably constructed based on official architectural improvement reports (e.g., efficiency enhancements brought by modules such as C3K2 and C2PSA) and typical trends from mainstream benchmark tests. The table aims to illustrate, both qualitatively and quantitatively, that YOLOv11 provides superior comprehensive performance over its predecessor in terms of accuracy, speed, and model efficiency, thereby justifying its selection as the core detection model in this study.

#### 2.2.4. Detection Workflow and System Integration

During operational deployment, the system performs panoramic scanning of meter-scale metal samples via a high-precision motion control platform. The trained YOLOv11 model enables real-time inclusion identification, localization, and adaptive segmentation. Detected targets are automatically logged with positional coordinates, and their classifications are verified either through laser spectral analysis or expert review. These results are further utilized to generate full-field inclusion distribution maps and size-frequency statistics, supporting quantitative assessment of material quality.

The system establishes a “detection-verification-optimization” closed-loop workflow, which not only ensures detection reliability but also significantly improves analytical efficiency. This provides an intelligent solution with continuous learning capabilities for quality control in metallic materials.

### 2.3. HSLA Steel Manufacturing Process and Sample Preparation

The production of HSLA steel constitutes a highly systematic and precision-engineered process, extending far beyond conventional steelmaking. It embodies an integrated “high-purity metallurgy” approach that combines multiple advanced technologies. The core objective is to minimize harmful elements and inclusions, ensure high uniformity in composition and microstructure, and thereby achieve an optimal balance of strength and toughness.

This study investigates a specific HSLA grade produced via the following state-of-the-art route: Electric Arc Furnace (EAF) → Ladle Furnace (LF) refining → Vacuum Degassing (VD) → Electroslag Remelting (ESR) → Ingot. The research systematically analyzes inclusion evolution throughout the multi-stage refining sequence: from a dia. 740 mm consumable electrode to a dia. 810 mm ESR ingot and finally to a dia. 400 mm forged billet. The composition range of the experimental steel is shown in Table 2.

To establish accurate material genetic correspondence, the sampling strategy designated the hot top and bottom of the original electrode as corresponding to the tail and head of the subsequent ESR ingot and final forged billet, respectively. Metallographic samples encompassing the radial direction were extracted from these corresponding locations to systematically study variations in the quantity, size, and distribution of inclusions throughout the entire processing route.

**Figure 2:**
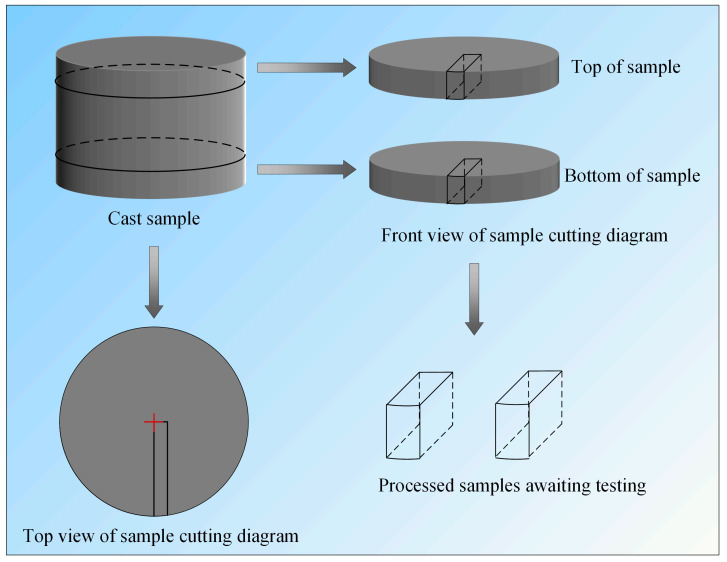
Schematic diagram of the HSLA sampling.

As illustrated in the sampling diagram (Figure 2), representative specimens were collected from different processing stages. Specifically:

Two samples each from the hot top and bottom of the dia. 740 mm consumable electrode.

Two samples each from the head and tail of the dia. 810 mm ESR ingot.

One sample each from the head and tail of the dia. 400 mm forged billet.

All samples were systematically labeled to facilitate subsequent data management and statistical analysis. The detailed sampling plan is summarized in Table 3. In this paper, “inner” and “outer” refer to regions near the geometric center and near the edge of the corresponding specimen, respectively.

### 2.4. Preparation of Large-Scale HSLA Steel Samples

To address the technical challenges associated with preparing meter-scale metallographic specimens, this study established a standardized preparation protocol based on a specialized grinding-polishing machine. Target samples were first sectioned using precision wire electrical discharge machining (EDM), with a 1–2 mm machining allowance reserved to ensure integrity and uniformity during subsequent grinding and polishing stages.

A large-scale grinding-polishing system was then employed for multi-stage processing: initial coarse grinding removed cutting marks, followed by fine grinding to refine surface morphology. Surface flatness was monitored in real time using a laser interferometer throughout the process.

A two-stage polishing procedure was implemented: rough polishing with 9 μm diamond suspension followed by fine polishing with 1 μm alumina suspension. This achieved a final surface roughness of Ra ≤ 0.02 μm, meeting the mirror-finish standard required for metallographic analysis. After polishing, samples underwent intensive ultrasonic cleaning with anhydrous ethanol to completely remove residual contaminants.

For quality assessment, white light interferometry was used to conduct full-field flatness inspection, and multiple randomly selected regions were examined at 100× magnification to ensure the absence of scratches, artifacts, or obscured areas. This standardized procedure is applicable to the industrial preparation of meter-scale specimens from materials such as steel and aluminum alloys. It significantly improves process consistency and preparation efficiency while ensuring forming accuracy, thereby providing a reliable specimen foundation for subsequent metallographic analysis.

## 3. Results

To establish a direct correlation between metallographic morphological classification (ASTM E45) and chemical composition analysis (LIBS), this study, based on the formation mechanisms and typical chemical compositions of inclusions, has clarified the characteristic elemental compositions corresponding to the four types of inclusions. The characteristic elements of Type A (sulfides) are Mn and S; Type B (aluminates) are centered around Al and often accompanied by Ca or Mg; the key identifier for Type C (silicates) is Si, which typically coexists with Mn; and Type D (globular oxides) manifest as combinations of O with one or more elements, such as Al, Mg, Ca, or Si, and generally lack signals for S and Mn. In practical analysis, the system first employs the YOLOv11 model for preliminary screening and localization based on morphological features, followed by triggering LIBS for in situ micro-area analysis of suspicious targets. This dual-mode strategy of “morphology-first, spectral-verification” significantly enhances the accuracy and reliability of automated inclusion classification and provides direct chemical evidence for understanding their evolution mechanisms.

In this study, non-metallic inclusions were classified and designated according to the ASTM E45 standard, systematically categorized by morphology, characteristics, and size differences into Type A (sulfides), Type B (aluminates), Type C (silicates), and Type D (globular oxides). Through automated identification and statistical analysis of valid metallographic images, multi-dimensional characteristic parameters of inclusions—including quantity, location, size, category, aspect ratio, and area—were obtained. To further verify the compositional makeup of inclusions, the top 100 largest inclusions identified through image analysis were selected for laser spectral analysis. This analysis detected the presence of metallic elements such as Mn, Al, Si, Mg, Ca, and Ti to clarify their chemical constitution. The laser spectral results were fed back in real time to the system database, supporting iterative training and optimization of the image recognition model for inclusion analysis across large-scale samples. Using the optimized model, inclusions were conclusively identified, and key information—including quantity, position, size, type, aspect ratio, and area—was statistically evaluated. A visualized inclusion distribution cloud map was also generated to comprehensively characterize the spatial distribution features of inclusions within the material.

**Figure 3:**
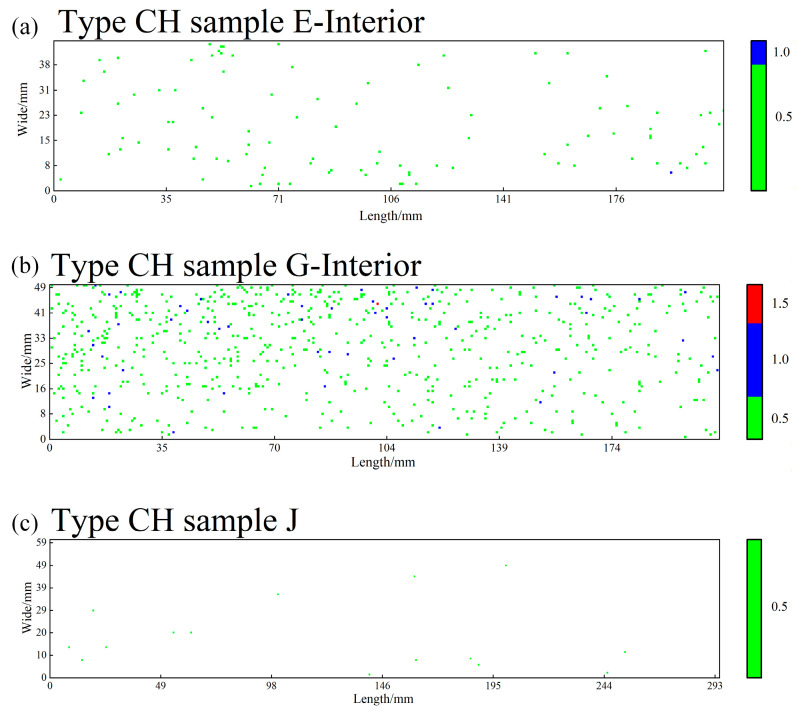
Enrichment and elimination process of Type C heavy series inclusions: (**a**) head of the ESR ingot, (**b**) tail of the ESR ingot, (**c**) tail of the forged billet.

**Figure 4:**
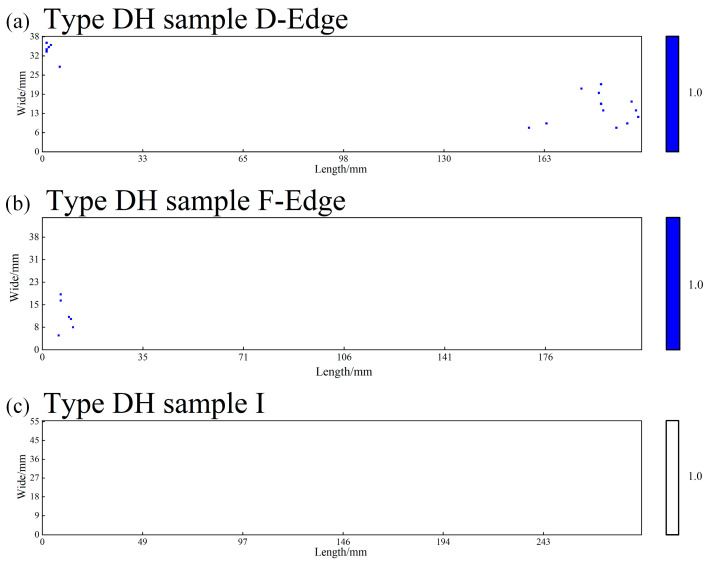
Continuous removal of Type D heavy series inclusions throughout the process: (**a**) tail of the consumable electrode, (**b**) head of the ESR ingot, (**c**) head of the forged billet.

**Figure 5:**
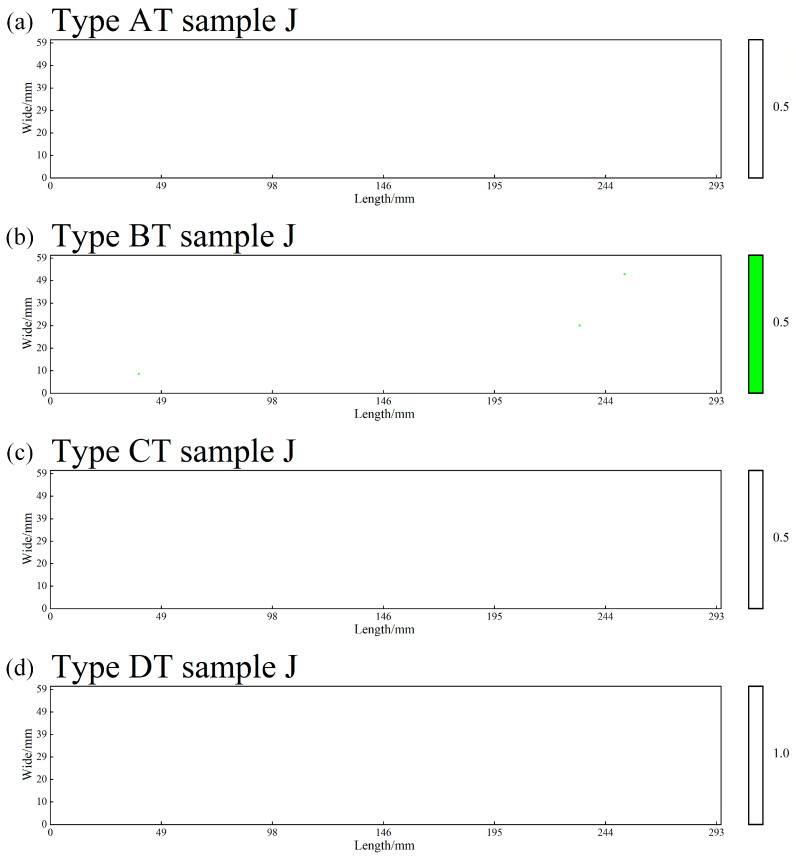
Rating distribution maps of the four types of thin series inclusions in the tail of the forged billet: (**a**) Type A thin series, (**b**) Type B thin series, (**c**) Type C thin series, (**d**) Type D thin series.

The “severity rating” referred to in this study denotes the digital assessment of the severity of inclusions within each field of view (0.5 mm^2^) in an automated analysis system, based on the fundamental principles of the ASTM E45 standard. The entire rating process is performed automatically by the system: first, the YOLOv11 model identifies and segments all inclusions in the field of view, automatically measuring their length (for Types A, B, and C) or quantity (for Type D). Subsequently, the system accumulates the measured values of inclusions of the same type within the same field of view according to their morphological classification. Next, the system’s built-in rating algorithm digitally maps the accumulated values to the ASTM E45 standard—for Type A (sulfides), Type B (aluminates), and Type C (silicates), the rating is determined based on the total length of all inclusions of the same type falling within the standard threshold intervals, converting it into corresponding “Thin (T)” or “Heavy (H)” series ratings (e.g., 0.5, 1.0, 1.5, etc.). For Type D (globular oxides), the rating is based on the total quantity distribution of all inclusions of the same type, determined in accordance with the standard. Finally, the system outputs a digital rating compliant with ASTM E45 semantics for each of the four inclusion types per field of view (e.g., AH: 1.0, CT: 0.5). All rating cloud maps (Figure 3, Figure 4 and Figure 5) and statistical tables (Table 4) presented in the paper are generated through this fully automated process.

Based on the metallographic analysis results, two-dimensional rating distribution maps were plotted for Type A, B, and C inclusions (based on cumulative length) and Type D inclusions (based on cumulative number) per 0.5 mm^2^ field of view. In these maps, the horizontal and vertical axes represent the two-dimensional spatial coordinates on the sample surface, while inclusion ratings are visualized using a color scale. The color gradient progresses from light to dark from bottom to top, corresponding to progressively higher inclusion severity levels. The designations “T” and “H” are used to indicate thin series and heavy series inclusions, respectively. Maps were generated separately for the heavy and thin series of each inclusion type (A, B, C, D). For Types A, B, and C, all inclusions with a severity level ≥ 0.5 were recorded, while for Type D, only those with a severity level ≥ 1.0 were counted. Table 4 provides a statistical summary of the number of fields categorized by inclusion type and severity rating. Figure 3 illustrates the “enrichment and elimination” evolution of Type C inclusions. This figure arranges the rating cloud maps for Type C (silicate) inclusions horizontally from three locations: the head of the ESR ingot, the tail of the ESR ingot, and the tail of the forged billet. It most intuitively presents a key finding of this study: the significant clustering of Type C inclusions in the ESR ingot tail (dense, dark-colored area) and their subsequent near-total elimination (returning to a sparse state) after forging, showing the complete process. Figure 4 shows the “continuous removal throughout the process” trend of Type D inclusions. This figure arranges the rating cloud maps for Type D (globular oxide) inclusions horizontally from three corresponding locations: the head of the consumable electrode, the head of the ESR ingot, and the head of the forged billet. It clearly demonstrates the continuous reduction trend of Type D inclusions—from their initial state, through removal during ESR, to reaching extremely low levels after forging—creating a visual impression of “order-of-magnitude reduction.” Figure 5 presents the “comprehensive purification effect” of the forging process. This figure focuses on the most representative location: the tail of the forged billet, displaying the rating cloud maps for all four types of inclusions (A, B, C, and D) at this site. The overall color scales in the four subpanels are extremely light. In particular, the distributions of Type A, B, and C inclusions are nearly absent, forming a stark contrast to the charts from the ESR ingot tail in the Supplementary Materials. This serves as the most direct visual evidence proving that “forging achieves exceptionally high material purity.” The inclusion rating contour maps for the head and tail positions along the “dia.740 mm consumable electrode → dia.810 mm ESR ingot → dia.400 mm forged billet” processing route are provided in Part II (Figures S1–S24) of the Supplementary Materials.

The distribution cloud maps clearly show that inclusions exhibit a non-uniform, clustered or banded distribution across the sample surface. In the tail section of the ESR ingot, Type C inclusions display a significantly high-density aggregation state (indicated by darker regions). This observation aligns with the statistical results, showing that the number of fields with Type C heavy series (CH) inclusions at the 0.5 grade level in this region reaches as high as 1211. In contrast, the inclusion distribution maps for the forged billet appear noticeably lighter in color overall, reflecting generally lower inclusion ratings and sparser distribution characteristics. Regarding process influence: a progressive homogenization of inclusion distribution and a reduction in severity ratings are observed from the consumable electrode to the ESR ingot and further to the forged billet. This trend reflects the effective role of ESR and forging processes in removing and refining inclusions.

Based on the statistical results of field counts (per 0.5 mm^2^) for various inclusions by sample location and severity grade in Table 4, the key findings are summarized as follows: In the feeder head of the dia. 740 mm consumable electrode, the field counts for Type A heavy series (AH) and Type C heavy series (CH) inclusions at grade 0.5 are 321 and 351, respectively, indicating that sulfides and silicates dominate in this region, with generally small sizes. Meanwhile, 79 fields with Type D heavy series (DH) inclusions at grade 1.0 were recorded, reflecting a certain distribution of globular oxides in this area. Overall, the number of higher-grade inclusions (1.5 and 2.0) is extremely low, suggesting effective control of inclusion sizes in this region.

In contrast, at the bottom end of the same consumable electrode, the counts of all inclusion types decrease, with the most significant reductions observed in Type A heavy series (from 321 to 260) and Type C heavy series (from 351 to 191). Only three fields with Type D heavy series at grade 1.0 were recorded, further confirming the overall lower inclusion levels in this area.

At the head of the dia. 810 mm ESR ingot, the field count for Type A heavy series (AH) at grade 0.5 reaches 425, with 60 and 5 fields at grades 1.0 and 1.5, respectively, indicating some aggregation of sulfides in this region. Type C heavy series (CH) has 288 fields at grade 0.5, but higher grades are limited. Type D heavy series (DH) at grade 1.0 shows 28 fields, representing a reduction compared to the consumable electrode.

At the tail of the ESR ingot, the field count for Type C heavy series (CH) at grade 0.5 increases sharply to 1211, with 103 and 7 fields at grades 1.0 and 1.5, respectively, demonstrating significant enrichment of silicate inclusions in the tail section. Meanwhile, Type A heavy series (AH) decreases to 243 fields at grade 0.5, and other inclusion types remain at low levels. This distribution characteristic is likely related to the flotation behavior of inclusions during ESR and solute segregation effects in the tail.

In the dia. 400 mm forged billet stage, the head shows a substantial reduction in all inclusion types. Only Type B heavy series (BH) and Type C heavy series (CH) remain at grade 0.5 with 53 and 64 fields, respectively, and no inclusions ≥ grade 1.0 are detected, indicating effective refinement and removal of inclusions by the forging process.

At the tail of the forged billet, inclusion counts decrease further, with only 21 and 15 fields for Type B heavy series (BH) and Type C heavy series (CH) at grade 0.5, respectively. No Type A or Type D inclusions are detected, reflecting the high purity achieved in this region.

In summary, the total number of fields with inclusions (with a rating ≥ 0.5) initially increased and then decreased from the consumable electrode to the ESR ingot. At the tail of the ESR ingot, the number of fields with Type C heavy series (CH) inclusions reached a peak of 1211, which is 3.2 times higher than that at the head (288 fields). After forging, the number of fields with high-rated inclusions (≥1.0) for all inclusion types dropped to zero, demonstrating effective inclusion control.

The aforementioned analysis indicates that both the ESR and forging processes effectively reduce the quantity and size of inclusions. The inclusion levels in the forged billet, particularly at the tail, are notably low, with the total number of Type A, B, and C inclusions reduced to 181 (Table 5), and no high-rated inclusions (≥Grade 1.0) observed (Table 4). This demonstrates a high degree of purity that meets the requirements for high-end applications. However, the pronounced aggregation of Type C inclusions in the tail of the ESR ingot requires attention, as their count reaches 1767, which is 3.9 times that of the head (Table 5).

According to the ASTM E45 standard, the inclusion ratings of all samples remain within acceptable limits. Nevertheless, significant differences are observed between the head and tail sections of both the consumable electrode and the ESR ingot, underscoring the need for location-specific inspection during production.

According to Table 5, throughout the entire manufacturing process of HSLA steel the quantity of non-metallic inclusions exhibits a systematic decreasing trend. Specifically, the total number of Type D inclusions decreases from approximately 10,000 in the consumable electrode to about 4000–7000 after ESR and further reduces to around 1000 after forging, representing an overall reduction of roughly one order of magnitude. This trend fully demonstrates the effective removal and refinement of inclusions achieved by the ESR and subsequent forging processes.

The most pronounced reduction in inclusion count occurs during the transition from the ESR ingot to the forged billet, indicating that hot deformation further promotes the fragmentation, dispersion, and even dissolution of inclusions.

Specifically, the removal of Type D (globular oxides) is particularly remarkable. Starting from very high levels in the consumable electrode (Feeder Head: 10,115; Bottom End: 8735), their numbers drop significantly after ESR (Head: 6898; Tail: 4052) and are further reduced to 1276 (Head) and 996 (Tail) in the forged billet. This reflects the efficient flotation and removal of globular oxides during ESR, especially in the ingot tail, where the prolonged solidification time favors inclusion flotation.

In contrast, Type C (silicates) shows significant enrichment in the tail section of the ESR ingot, with the count surging from 358 in the head to 1767 in the tail. This aligns with the described segregation behavior of silicates, likely attributable to their lower density causing slower flotation and their aggregation in the final solidification zone due to solute redistribution.

The quantities of Type A (sulfides) and Type B (aluminates) decrease progressively throughout the process. Type A inclusions, initially around 3000 in the consumable electrode, decrease slightly after ESR and further drop to 896 (Head) and 135 (Tail) after forging. Type B inclusions, generally lower in number, continue to decrease after forging, indicating that the forging process also contributes to the fragmentation and dispersion of hard alumina-type inclusions.

At the tail of the dia. 400 mm forged billet, the quantities of all types of inclusions are reduced to relatively low levels (Table 5). Among these, the combined total of Type A, B, and C inclusions is only 181, and no fields with a rating ≥ 1.0 are observed (Table 4), demonstrating that both the size and quantity of inclusions have been effectively controlled. This improvement contributes to the enhanced service reliability of components manufactured in this region.

By integrating data from the figures and Table 4, this analysis comprehensively characterizes the distribution and behavior of inclusions in the HSLA samples, providing a scientific basis for further process improvement and quality control.

Systematic Recommendations for Enhanced Cleanliness and Performance of HSLA Steels, Based on a systematic analysis of the distribution characteristics of non-metallic inclusions in HSLA materials, the following integrated recommendations are proposed to further improve material cleanliness and overall performance: To address the pronounced clustering of Type C silicate inclusions in the tail of the ESR ingot, process-level optimizations should be implemented at the source. This includes adjusting melting parameters to form a shallow molten pool, adopting highly adsorptive slag systems, and strictly controlling the content of [Si], [Al], and [O] to effectively suppress inclusion segregation. The optimization mechanism lies in: (1) shortening the length of the mushy zone to reduce solute enrichment during the final solidification stage, thereby suppressing the formation of silicates at their source, and (2) reducing the distance for inclusions to float to the slag–metal interface and lowering their probability of being captured by dendrites, thereby promoting the absorption of inclusions by the molten slag before solidification. This “shallow molten pool” control strategy represents a key optimization direction for addressing the cleanliness issue in the ingot tail. It aims to systematically improve the inclusion control capability of the ESR process by optimizing the mass and heat transfer conditions at the solidification front. A standardized sampling protocol should be established for key locations throughout the entire production route. A multi-dimensional monitoring system—encompassing type distribution, thin/heavy series ratio, and spatial morphology—should be implemented to accurately identify process bottlenecks. Furthermore, a data-driven approach should be deepened through the construction of a digital twin system that integrates spectral composition and metallographic characteristics. This will facilitate a shift in quality control from post-event inspection to predictive modeling. Finally, the beneficial effect of the forging process on inclusion refinement should be consolidated. In-depth studies on the deformation behavior of different inclusion types during hot working and their impact on material anisotropy are recommended. By implementing this full-process precision control system—spanning melting, remelting, and forging—material cleanliness and performance consistency can be significantly enhanced, providing reliable support for the manufacture of high-end HSLA materials.

**Figure 6:**
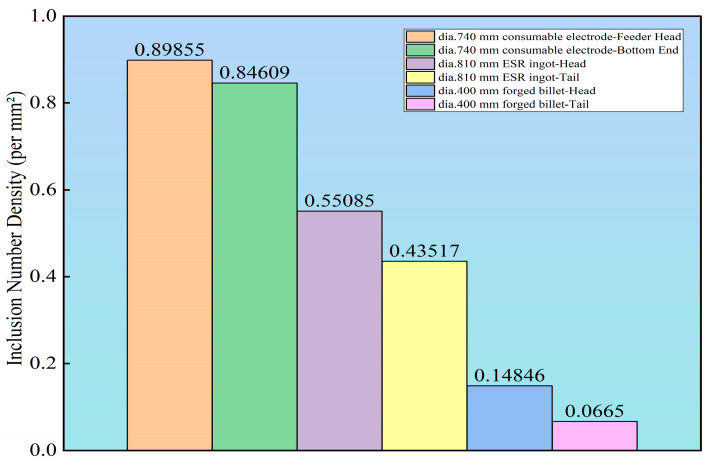
Inclusion Number Density.

Figure 6 is highly consistent with the spatial distribution characteristics displayed in the rating cloud maps. In the tail of the ESR ingot, although Table 5 indicates that the total number of Type C inclusions reaches 1767, the rating cloud maps reveal significant clustered aggregation of Type C inclusions in this region. This localized enrichment directly leads to the abnormally high number density value observed there. Furthermore, data from Table 4 show that in the tail of the ESR ingot, Type C heavy series (CH) inclusions reach 103 and 7 fields at grades 1.0 and 1.5, respectively, confirming that this region contains not only a large quantity of inclusions but also inclusions with higher severity ratings. In contrast, the forged billet exhibits a significantly reduced number density, particularly in the tail section, where it reaches an extremely low level. This is consistent with the light-colored, uniform distribution shown in the corresponding rating cloud maps.

**Figure 7:**
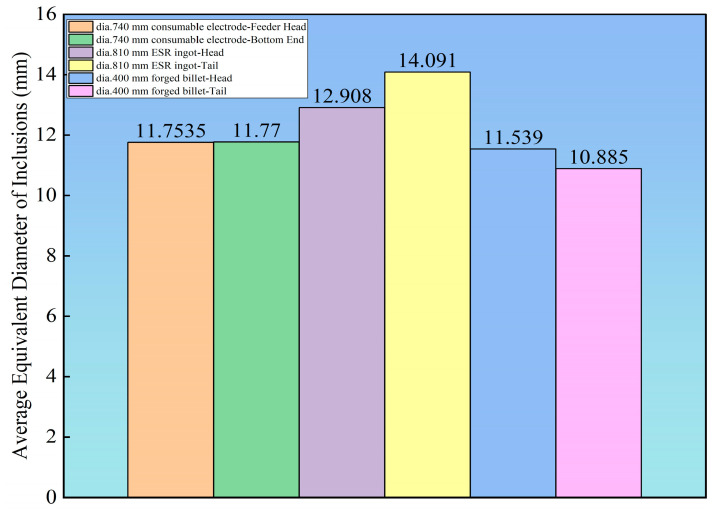
Average Equivalent Diameter of Inclusions.

Figure 7 demonstrates a strong correlation between the evolution of inclusion size and the field count statistics by severity rating. During the ESR process, although the total number of Type D (globular oxides) decreased from approximately 10,000 in the consumable electrode to between 4000 and 7000 in the ESR ingot (Table 5), the average equivalent diameter may have increased due to the preferential flotation of smaller inclusions. This observation aligns with the relative stability in the number of fields rated at grade 1.0 for Type D heavy series (DH) inclusions shown in Table 4. A significant reduction in the average equivalent diameter was achieved during the forging stage. Particularly in the tail of the forged billet, Table 3 shows that the quantities of all inclusion types dropped to very low levels, while the corresponding average equivalent diameter in Figure 7 also reached its minimum. This confirms that the forging process effectively refined the inclusion sizes, which is fully consistent with the fine and dispersed distribution characteristics displayed in the rating cloud maps.

The significant refinement of inclusion size during forging is primarily attributed to the synergistic interplay between mechanical and thermodynamic effects under hot deformation conditions: (1) For brittle oxides (Types B and D), strain mismatch between the matrix and the inclusions induces stress concentration, leading to brittle fracture; (2) For plastic sulfides and some silicates (Types A and C), they undergo plastic elongation, necking, and subsequent fracture into smaller units; (3) Migration of dynamic recrystallization grain boundaries in the matrix can strip or fragment inclusions, promoting their dispersion; (4) High temperature and deformation-driven diffusion accelerate the interfacial dissolution of small-sized or soluble inclusions, especially certain silicates. These mechanisms collectively contribute to the reduction in the average equivalent diameter of all inclusion types after forging (Figure 7) and their more uniform distribution (Figure 5), ultimately achieving a substantial improvement in material cleanliness.

## 4. Conclusions

This study integrates high-precision large-scale detection technology with in-depth data analysis to systematically reveal the hereditary evolution pattern of non-metallic inclusions in HSLA steel throughout the entire process chain of “consumable electrode → ESR ingot → forged billet.” It provides a solid theoretical foundation and advanced tool support for achieving precise and intelligent quality control of “defects known and controllable,” holding significant engineering importance for advancing the development and application of key component materials in high-end equipment.

The research clarifies the evolution mechanisms and quantitative trends of inclusions across the full process. The ESR process demonstrates significant removal capability for Type D (globular oxides), with their quantity reduced by approximately one order of magnitude, primarily attributed to the synergistic effects of slag adsorption and bubble flotation. However, in the ingot tail, due to solute redistribution from terminal solidification competing with inclusion flotation dynamics, the quantity of Type C (silicates) increases sharply to 3.9 times that in the head, forming localized enrichment. The subsequent forging process further promotes the fragmentation, dispersion, and even dissolution of all inclusion types through thermo-mechanical action. Particularly in the billet tail, the total number of Type A, B, and C inclusions is reduced to 181, with no inclusions rated ≥ 1.0, achieving exceptionally high material purity and thereby significantly enhancing the service reliability of components.

Methodologically, the self-developed large-scale, full-range analysis system—integrating microscopic matrix imaging, laser-induced breakdown spectroscopy (LIBS), and GPU-accelerated computing—overcomes the statistical limitations of traditional metallographic sampling. It enables rapid, fully automated identification and compositional analysis of inclusions in meter-scale samples. This facilitates a fundamental paradigm shift in quality assessment from “meeting smelting specifications” toward “defects known and controllable.”

At the analytical capability level, the intelligent detection framework integrating the YOLOv11 deep learning model with LIBS spectral verification achieves high-precision and high-efficiency classification of the four inclusion types. The established “detection-verification-optimization” closed-loop workflow forms an analytical system with continuous learning capabilities, laying the technical foundation for constructing a material digital twin system and enabling proactive process control.

Based on the above findings and technological achievements, this study further proposes systematic process optimization strategies. During the ESR stage, measures such as controlling molten pool morphology (e.g., shallow pool), optimizing slag composition, and strictly regulating the contents of [Si], [Al], and [O] should be implemented to suppress silicate segregation in the tail. During the forging stage, its excellent capability for inclusion refinement and dispersion should be consolidated and utilized. Finally, by establishing a monitoring system encompassing key location sampling and multi-dimensional data analysis throughout the entire process, a comprehensive solution is formed to enhance the cleanliness and performance consistency of HSLA steel, providing reliable assurance for the stable production of materials for high-end applications.

While the integrated framework demonstrated robust performance in this study, we acknowledge that the initial scale of the manually annotated dataset presents a constraint. The model’s generalizability to entirely new steel grades or radically different inclusion types formed under novel process regimes requires further validation. Future work will focus on: (1) systematically expanding the labeled dataset across a broader range of HSLA grades and process conditions; (2) exploring semi-supervised learning techniques to leverage vast amounts of unlabeled metallographic images; and (3) conducting rigorous external validation trials in industrial partner facilities. These steps will further solidify the framework’s robustness and pave the way for its broader adoption as a standard tool for intelligent cleanliness control.

## Figures and Tables

**Table 1:** Performance comparison between YOLOv11 and YOLOv8.

Model	mAP@0.5 (%)	Inference Speed (FPS) on T4	Small-Target Recall (<20 px) (%)	Applicability and Remarks
YOLOv11	92.5	140	93.2	End-to-end; optimal balance of accuracy, speed, and efficiency; meets the requirements for high-throughput real-time detection and industrial deployment.
YOLOv8	90.4	115	91.5	Powerful performance, but slightly inferior to v11 in structural efficiency and latest optimizations.

**Table 2:** HSLA Steel Composition Range.

Element	C	Si	Mn	Cr	Mo	Ni	V	P	S
Content (Wt%)	0.40–0.44	1.45–1.80	0.60–0.90	0.65–0.95	0.30–0.45	1.60–2.00	0.05–0.10	≤0.010	≤0.008

**Table 3:** Sampling scheme of the consumable electrode, ESR ingot, and forged billet.

Specifications of Materials	Sampling Location	Specimen	Specimen Number	Sample Dimensions (mm)
dia. 740 mm consumable electrode	Feeder Head	dia. 740 mm—interior	A	40 × 190
		dia. 740 mm—Edge	B	40 × 190
	Bottom End	dia. 740 mm—interior	C	38 × 195
		dia. 740 mm—Edge	D	38 × 195
dia. 810 mm ESR (Electroslag Remelting) ingot	Head	dia. 810 mm—interior	E	45 × 210
		dia. 810 mm—Edge	F	45 × 210
	Tail	dia. 810 mm—interior	G	50 × 207
		dia. 810 mm—Edge	H	50 × 207
dia. 400 mm forged billet	Head	dia. 400 mm	I	55 × 290
	Tail	dia. 400 mm	J	60 × 295

**Table 4:** Statistics of Field Counts by Inclusion Type and Rating Level.

Specimen Number	Inclusion Rating	AT	AH	BT	BH	CT	CH	DT	DH
dia. 740 mm consumable electrode—Feeder Head	0.5	0	321	36	57	6	351		
	1.0	0	21	2	0	0	2	0	79
	1.5	0	1	0	0	0	1	0	2
	2.0	0	1	0	0	0	0	0	0
dia. 740 mm consumable electrode—Bottom End	0.5	3	260	48	64	5	191		
	1.0	0	9	0	0	0	0	0	3
	1.5	0	0	0	0	0	0	0	0
	2.0	0	0	0	0	0	0	0	0
dia. 810 mm ESR ingot—Head	0.5	8	425	29	34	5	288		
	1.0	0	60	0	0	0	5	0	28
	1.5	0	5	0	0	0	0	0	0
	2.0	0	0	0	0	0	0	0	0
dia. 810 mm ESR ingot—Tail	0.5	1	243	24	21	8	1211		
	1.0	0	5	0	0	0	103	0	3
	1.5	0	0	0	0	0	7	0	0
	2.0	0	0	0	0	0	0	0	0
dia. 400 mm forged billet—Head	0.5	0	10	20	53	0	64		
	1.0	0	0	0	0	0	0	0	0
	1.5	0	0	0	0	0	0	0	0
	2.0	0	0	0	0	0	0	0	0
dia. 400 mm forged billet—Tail	0.5	0	1	3	21	0	15		
	1.0	0	0	0	0	0	0	0	0
	1.5	0	0	0	0	0	0	0	0
	2.0	0	0	0	0	0	0	0	0

**Table 5:** Variation in Inclusion Counts Across Different Processing Stages.

Specimen Number	A	B	C	D
dia. 740 mm consumable electrode—Feeder Head	2854	217	472	10,115
dia. 740 mm consumable electrode—Bottom End	3322	201	281	8735
dia. 810 mm ESR ingot—Head	3047	108	358	6898
dia. 810 mm ESR ingot—Tail	3000	189	1767	4052
dia. 400 mm forged billet—Head	896	100	96	1276
dia. 400 mm forged billet—Tail	135	30	16	996

## Data Availability

The data presented in this study are available on request from the corresponding author due to the study is ongoing.

## Supplementary Materials

The following supporting information can be downloaded at: https://www.mdpi.com/article/10.3390/ma19010158/s1, Figure S1: Inclusion rating distribution of Type A inclusions (H and T series) in the dia.740 mm consumable electrode—Feeder Head; Figure S2: Inclusion rating distribution of Type B inclusions (H and T series) in the dia.740 mm consumable electrode—Feeder Head; Figure S3: Inclusion rating distribution of Type C inclusions (H and T series) in the dia.740 mm consumable electrode—Feeder Head; Figure S4: Inclusion rating distribution of Type D inclusions (H and T series) in the dia.740 mm consumable electrode—Feeder Head; Figure S5: Inclusion rating distribution of Type A inclusions (H and T series) in the dia.740 mm consumable electrode—Bottom End; Figure S6: Inclusion rating distribution of Type B inclusions (H and T series) in the dia.740 mm consumable electrode—Bottom End; Figure S7: Inclusion rating distribution of Type C inclusions (H and T series) in the dia.740 mm consumable electrode—Bottom End; Figure S8: Inclusion rating distribution of Type D inclusions (H and T series) in the dia.740 mm consumable electrode—Bottom End; Figure S9: Inclusion rating distribution of Type A inclusions (H and T series) in the dia.810 mm ESR ingot—Head; Figure S10. Inclusion rating distribution of Type B inclusions (H and T series) in the dia.810 mm ESR ingot—Head; Figure S11. Inclusion rating distribution of Type C inclusions (H and T series) in the dia.810 mm ESR ingot—Head; Figure S12: Inclusion rating distribution of Type D inclusions (H and T series) in the dia.810 mm ESR ingot—Head; Figure S13: Inclusion rating distribution of Type A inclusions (H and T series) in the dia.810 mm ESR ingot—Tail; Figure S14: Inclusion rating distribution of Type B inclusions (H and T series) in the dia.810 mm ESR ingot—Tail; Figure S15: Inclusion rating distribution of Type C inclusions (H and T series) in the dia.810 mm ESR ingot—Tail; Figure S16: Inclusion rating distribution of Type D inclusions (H and T series) in the dia.810 mm ESR ingot—Tail; Figure S17: Inclusion rating distribution of Type A inclusions (H and T series) in the dia.400 mm forged billet—Head; Figure S18: Inclusion rating distribution of Type B inclusions (H and T series) in the dia.400 mm forged billet—Head; Figure S19: Inclusion rating distribution of Type C inclusions (H and T series) in the dia.400 mm forged billet—Head; Figure S20: Inclusion rating distribution of Type D inclusions (H and T series) in the dia.400 mm forged billet—Head; Figure S21: Inclusion rating distribution of Type A inclusions (H and T series) in the dia.400 mm forged billet—Tail; Figure S22: Inclusion rating distribution of Type B inclusions (H and T series) in the dia.400 mm forged billet—Tail; Figure S23: Inclusion rating distribution of Type C inclusions (H and T series) in the dia.400 mm forged billet—Tail; Figure S24: Inclusion rating distribution of Type D inclusions (H and T series) in the dia.400 mm forged billet—tail.
