# Supplementary material for: A Machine Vision-Enhanced Framework for Tracking Inclusion Evolution and Enabling Intelligent Cleanliness Control in Industrial-Scale HSLA Steels
Source: Materials (Basel). 2026 Jan 2;19(1):158. doi: 10.3390/ma19010158 (PMC12786806; doi:10.3390/ma19010158)
Supplement: Supplementary file 1 [file materials-19-00158-s001.zip › materials-4047571-supplementary.pdf]

materials

Article

A Machine Vision-Enhanced Framework for Tracking Inclusion Evolution and Enabling Intelligent Cleanliness Control in Industrial-Scale HSLA Steels

Yong Lyu [1,2], Yunhai Jia [2,*], Lixia Yang [2],Weihao Wan [2], Danyang Zhi [2], Xuehua Wang [2], Peifeng Cheng [2] and Haizhou Wang [2]

[1] School of Metallurgical and Ecological Engineering, University of Science and Technology Beijing, Beijing 100083,China; 15332728942@163.com
[2] Central Iron & Steel Research Institute, Beijing 100081,China
* Correspondence: jiayunhai@ncschina.com

1. Part I Instrumentation and Operating Parameters

The analytical system comprises four core modules, which are technically coordinated in both parameters and functionality to achieve high-throughput, automated characterization of inclusions in meter-scale metallic samples.

1.High-Precision CNC Stage

The positioning stage is constructed with a high-rigidity marble base and employs a closed-loop servo control system. Its overall dimensions are 2500 mm × 2280 mm × 3270 mm, with travel ranges of 1000 mm (X-axis), 500 mm (Y-axis), and 500 mm (Z-axis). Capable of handling heavy, meter-scale metallographic samples, the stage achieves micrometer-level motion precision. It demonstrates a bidirectional positioning accuracy better than 2 μm over a 700 mm travel distance and a repeat positioning accuracy of ≤1 μm over 100 mm, as validated by 20 reciprocating tests. This level of precision ensures pixel-level registration consistency at a 100x magnification and simultaneously meets the stringent positioning requirements for in-situ micro-scale laser spot spectral (LIBS) detection.

2. Microscopic Matrix Imaging System

The system integrates 24 microscopic imaging units arranged in a 4×6 matrix, with each unit responsible for capturing images from a 120 mm × 120 mm area. Under 100x magnification, the effective imaging area for a single field of view (FOV) is 1.5 mm × 1.5 mm. This configuration enables parallel and high-speed image capture over large-scale samples.

3. Laser Spectral Analysis System

The system is equipped with an Nd:YAG-pumped solid-state laser operating at a 1064 nm wavelength, with a pulse duration of <9 ns and a beam divergence of <1.5 mrad. The focused laser produces a spot size of 30 μm. Under software control, the system performs automatic focusing and spectral signal acquisition for qualitative identification of inclusion composition.

4. GPU High-Performance Computing Cluster

The system is centered around a GPU computing cluster, where a master server is connected to six subordinate workstations via a network switch, enabling centralized scheduling of image acquisition, motion control, and spectral analysis. A dedicated image processing software, developed on GPU-parallelized algorithms, performs real-time image processing and inclusion identification concurrently with data acquisition. This includes morphology extraction, position calibration, and spectral data analysis, ultimately

achieving rapid localization and compositional statistics of inclusions across full-scale samples.

2. Part II Full-process inclusion rating contour maps.

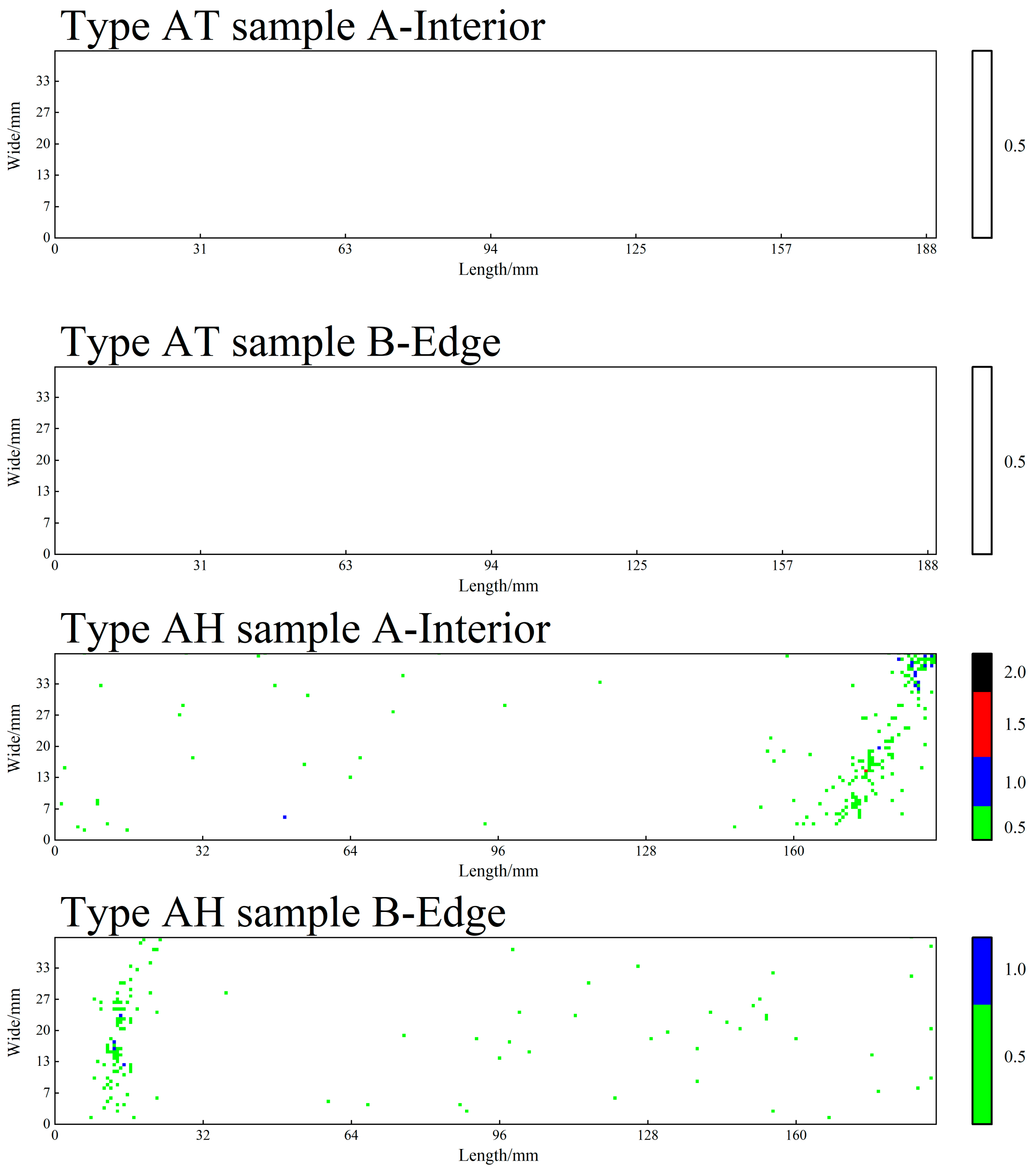

Figure S1. Inclusion rating distribution of Type A inclusions (H and T series) in the dia.740 mm consumable electrode - Feeder Head.

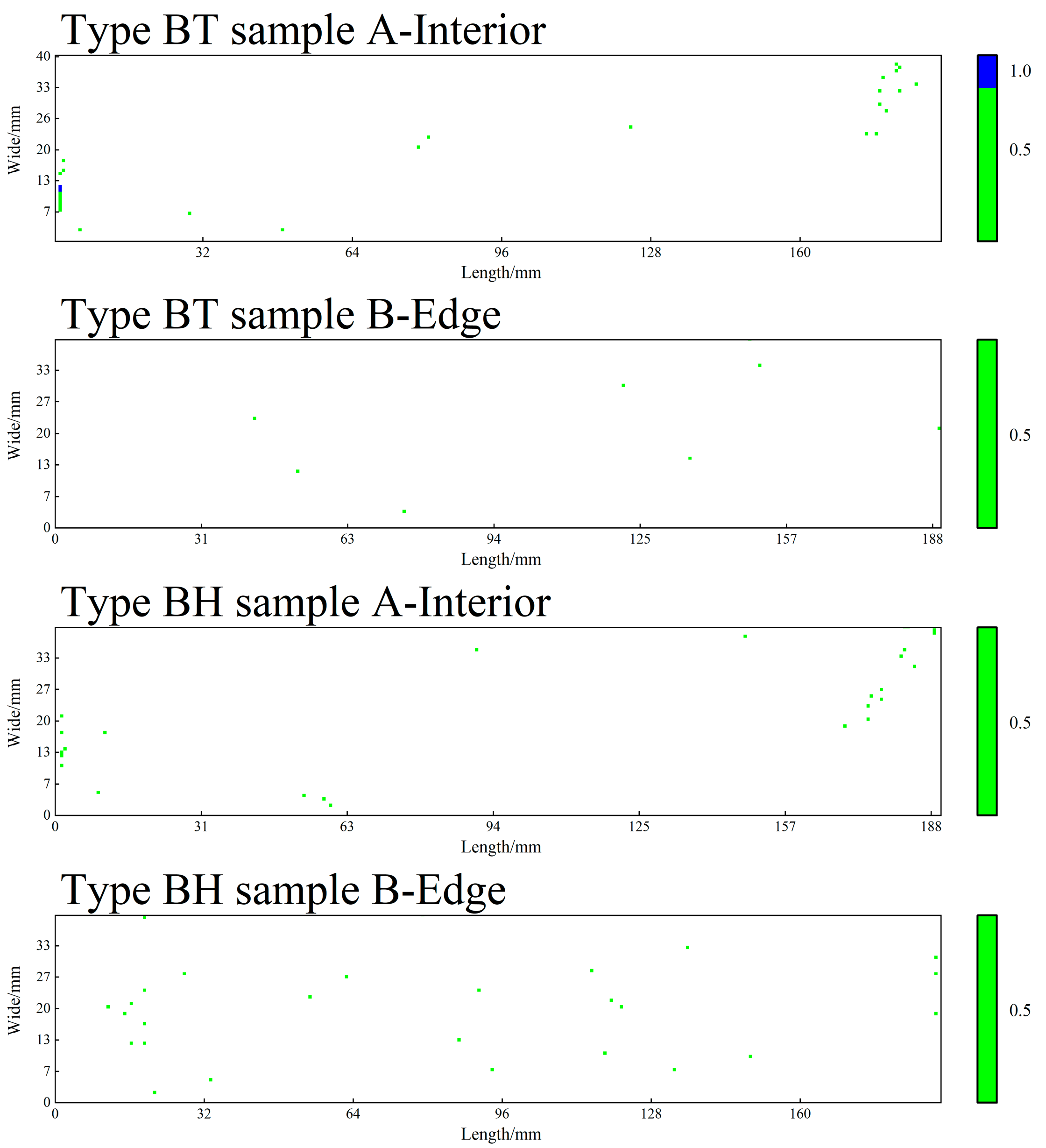

Figure S2. Inclusion rating distribution of Type B inclusions (H and T series) in the dia.740 mm consumable electrode - Feeder Head.

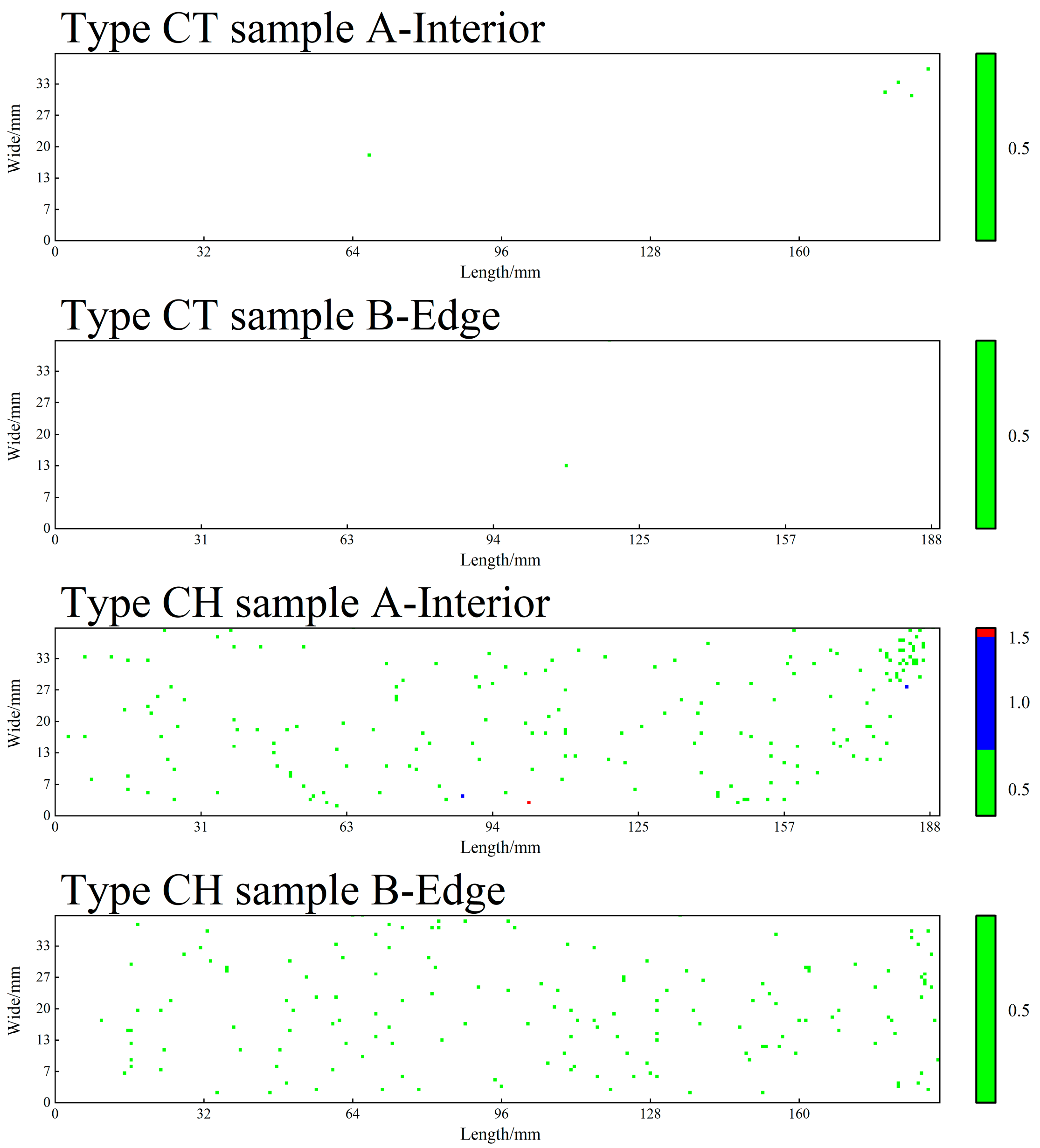

Figure S3. Inclusion rating distribution of Type C inclusions (H and T series) in the dia.740 mm consumable electrode - Feeder Head.

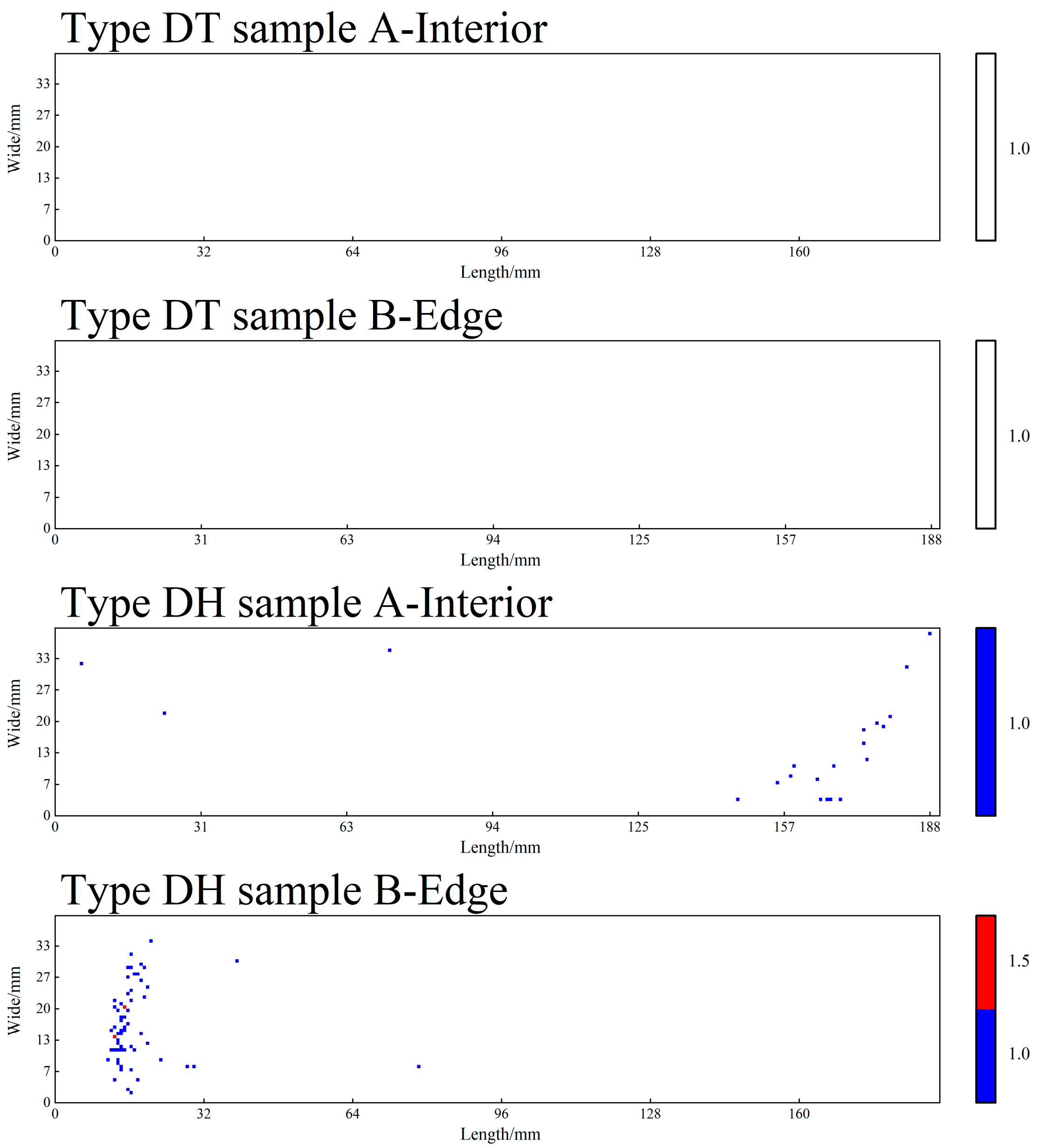

Figure S4. Inclusion rating distribution of Type D inclusions (H and T series) in the dia.740 mm consumable electrode - Feeder Head.

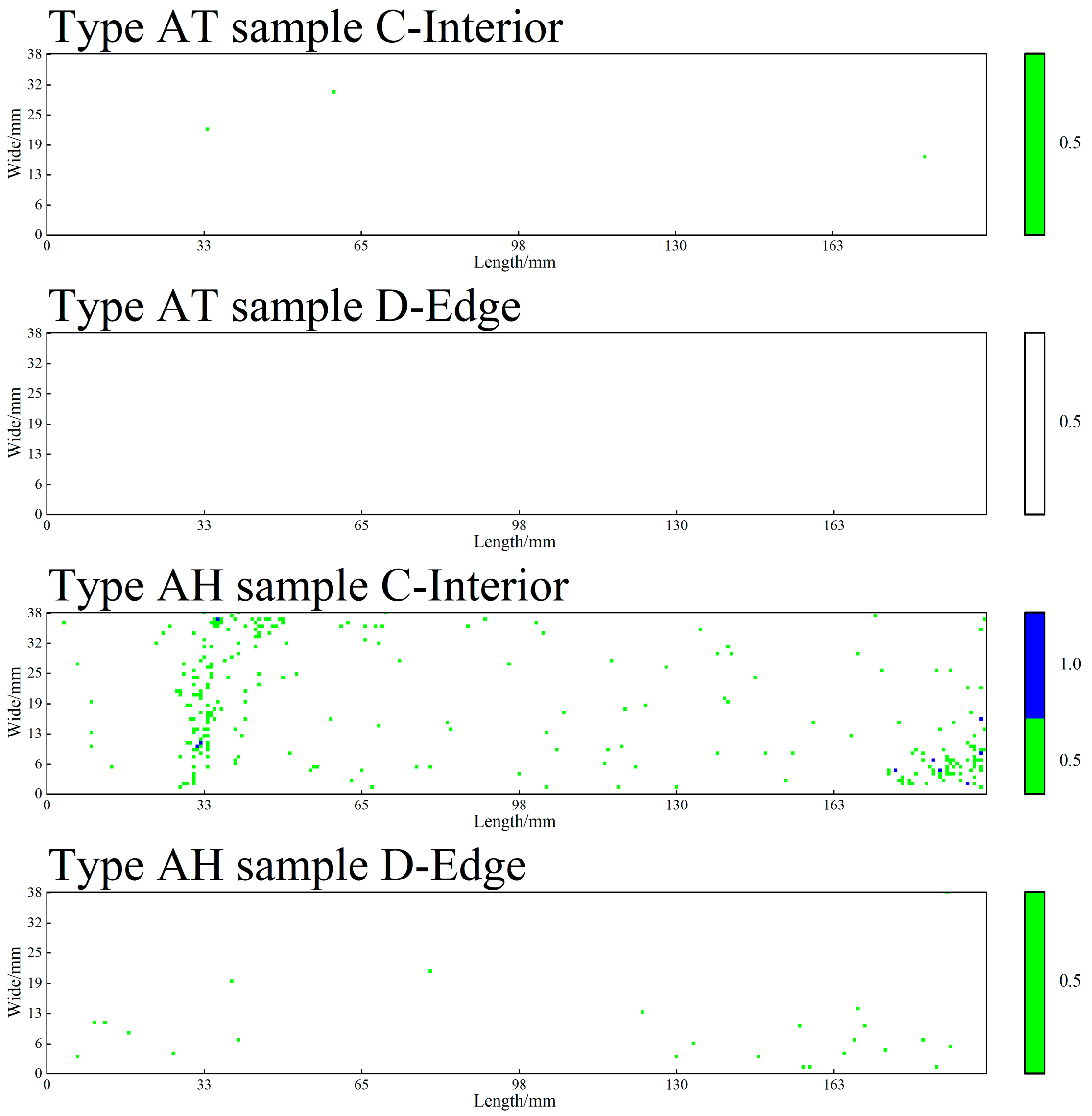

Figure S5. Inclusion rating distribution of Type A inclusions (H and T series) in the dia.740 mm consumable electrode - Bottom End.

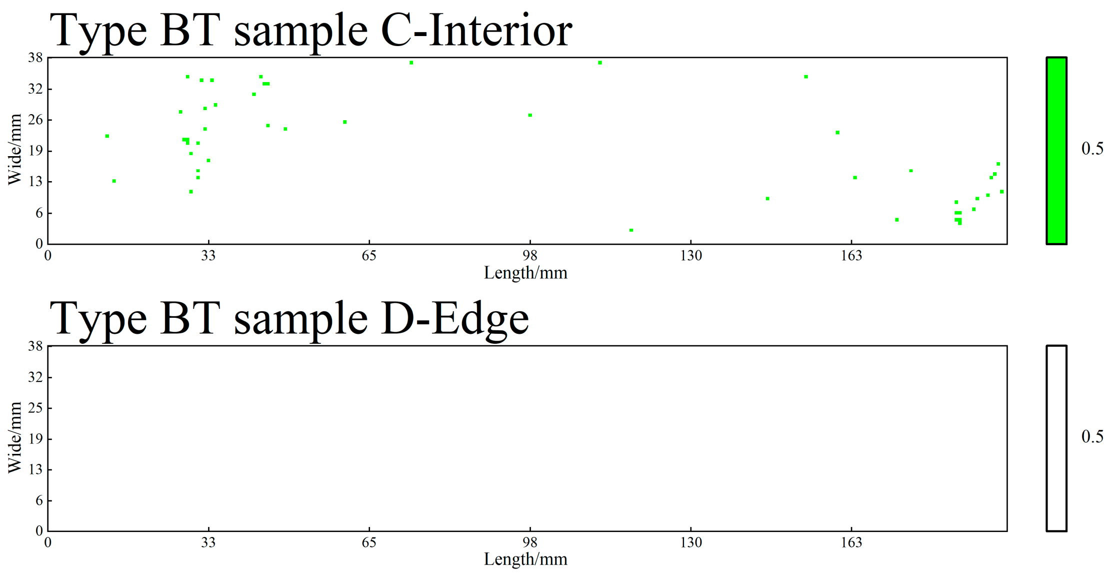

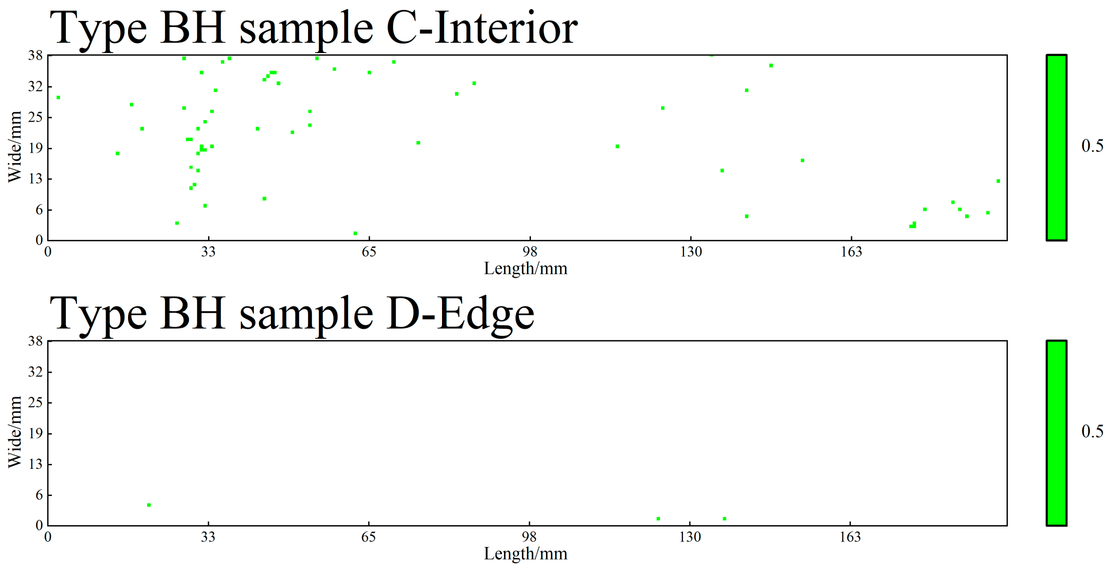

Figure S6. Inclusion rating distribution of Type B inclusions (H and T series) in the dia.740 mm consumable electrode - Bottom End.

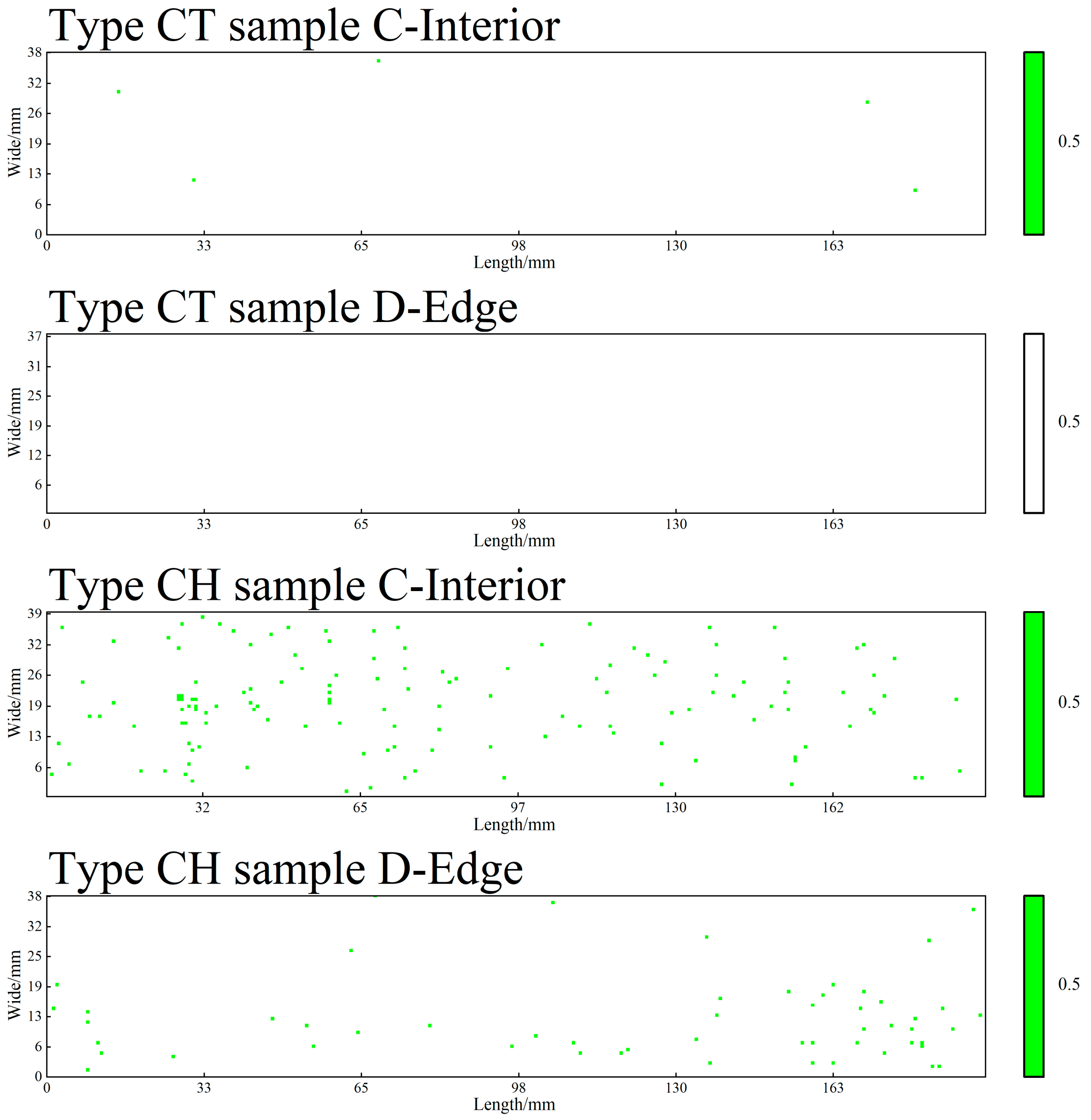

Figure S7. Inclusion rating distribution of Type C inclusions (H and T series) in the dia.740 mm consumable electrode - Bottom End.

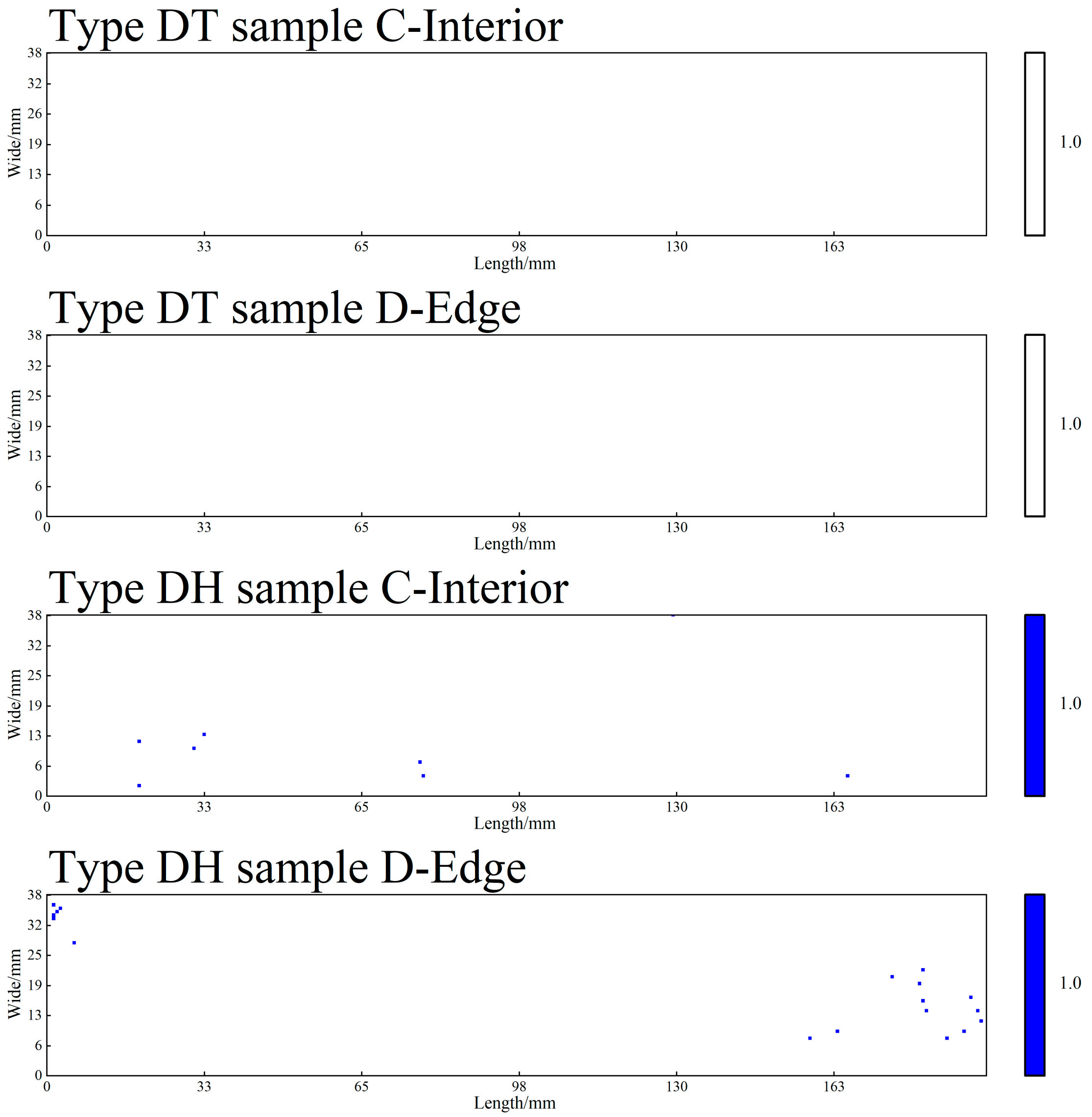

Figure S8. Inclusion rating distribution of Type D inclusions (H and T series) in the dia.740 mm consumable electrode - Bottom End.

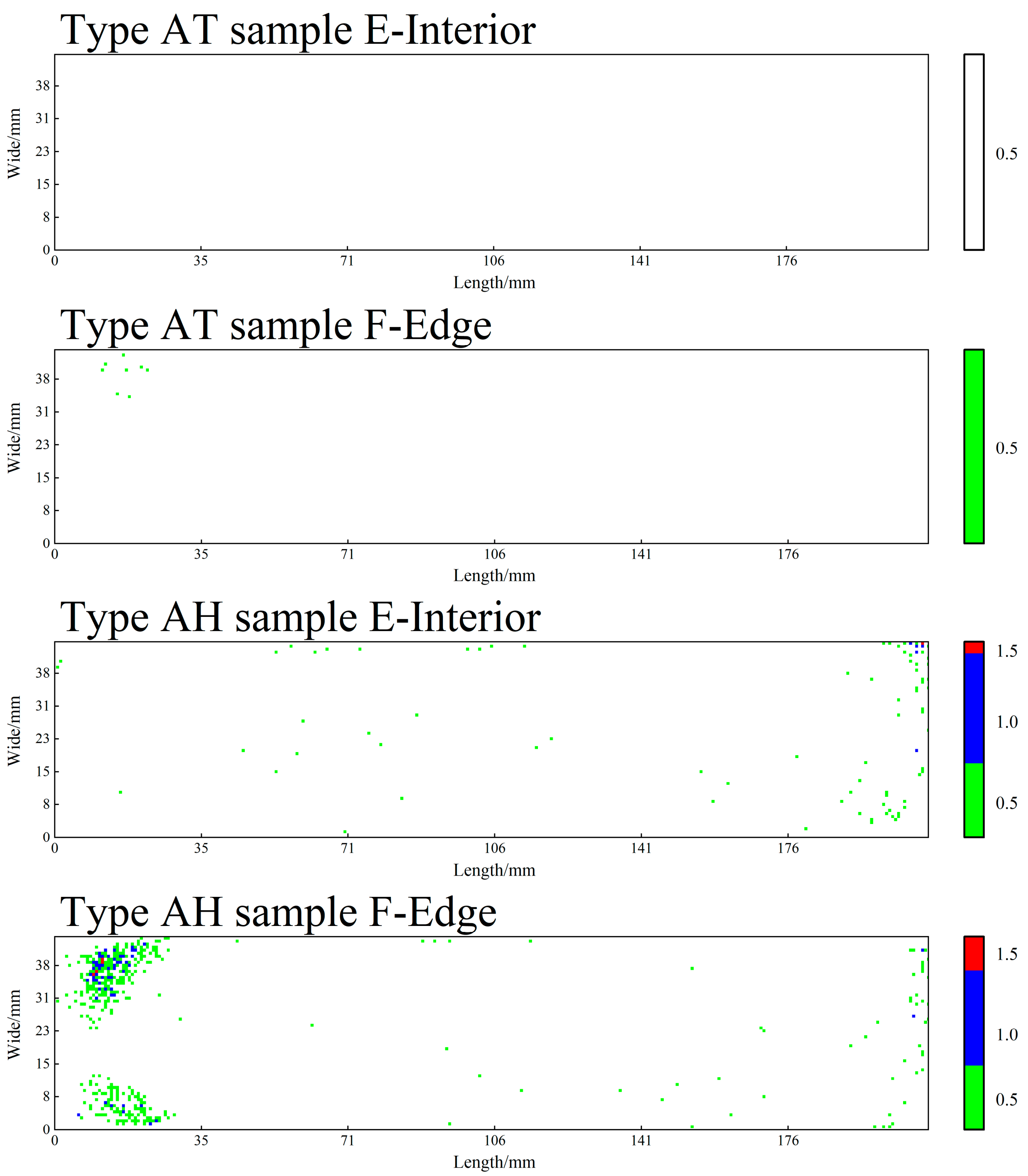

Figure S9. Inclusion rating distribution of Type A inclusions (H and T series) in the dia.810 mm ESR ingot-Head.

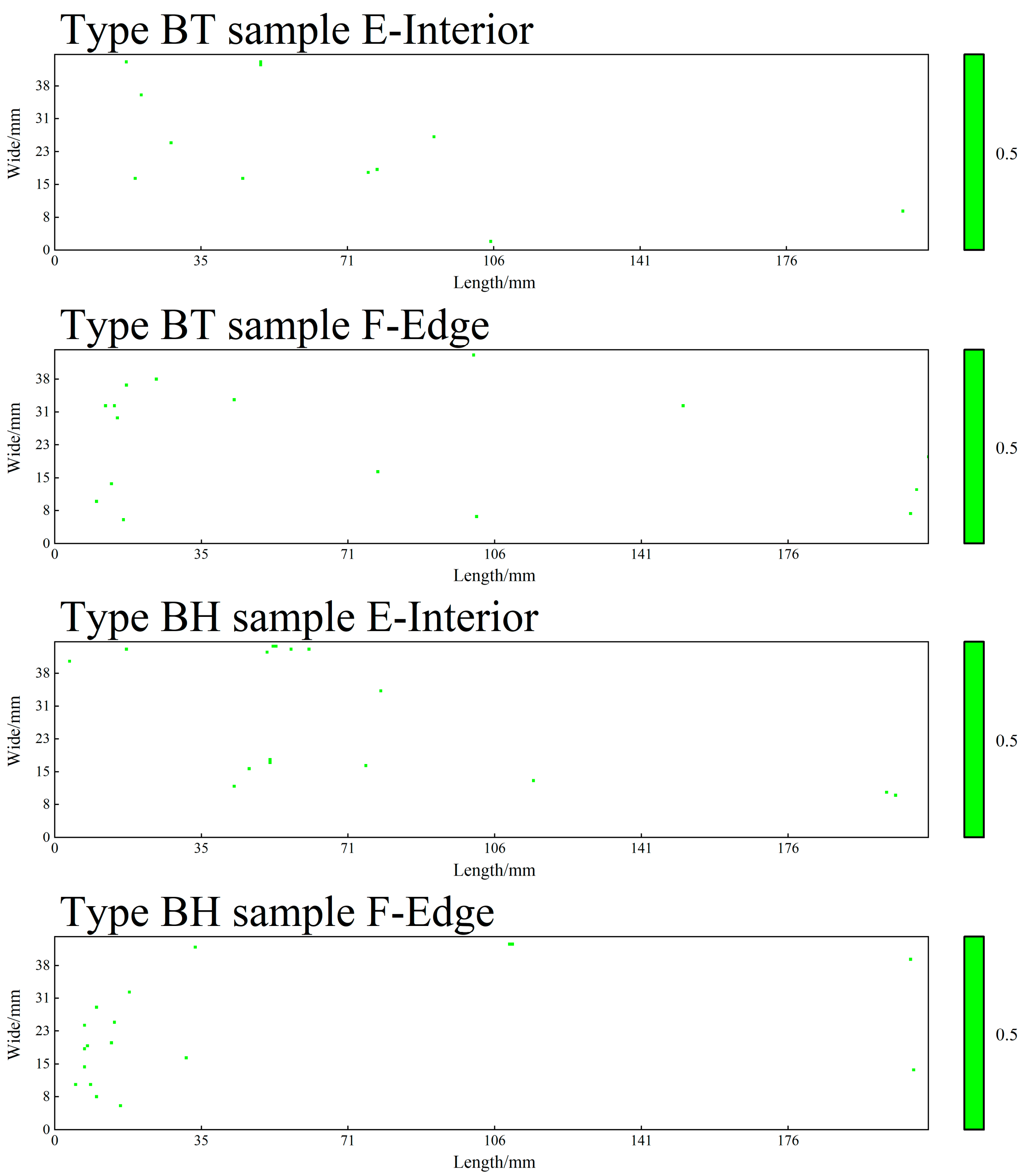

Figure S10. Inclusion rating distribution of Type B inclusions (H and T series) in the dia.810 mm ESR ingot-Head.

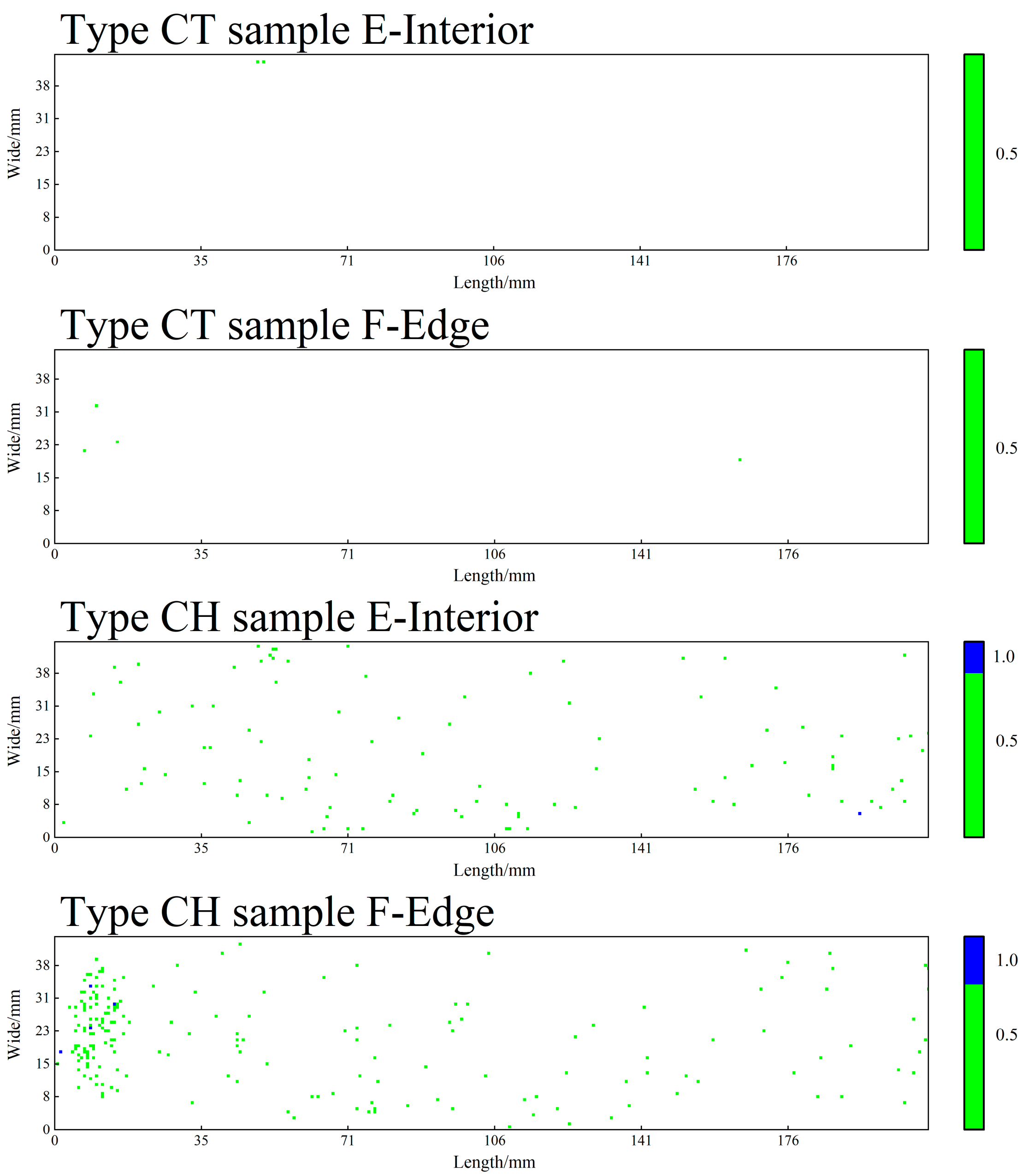

Figure S11. Inclusion rating distribution of Type C inclusions (H and T series) in the dia.810 mm ESR ingot-Head.

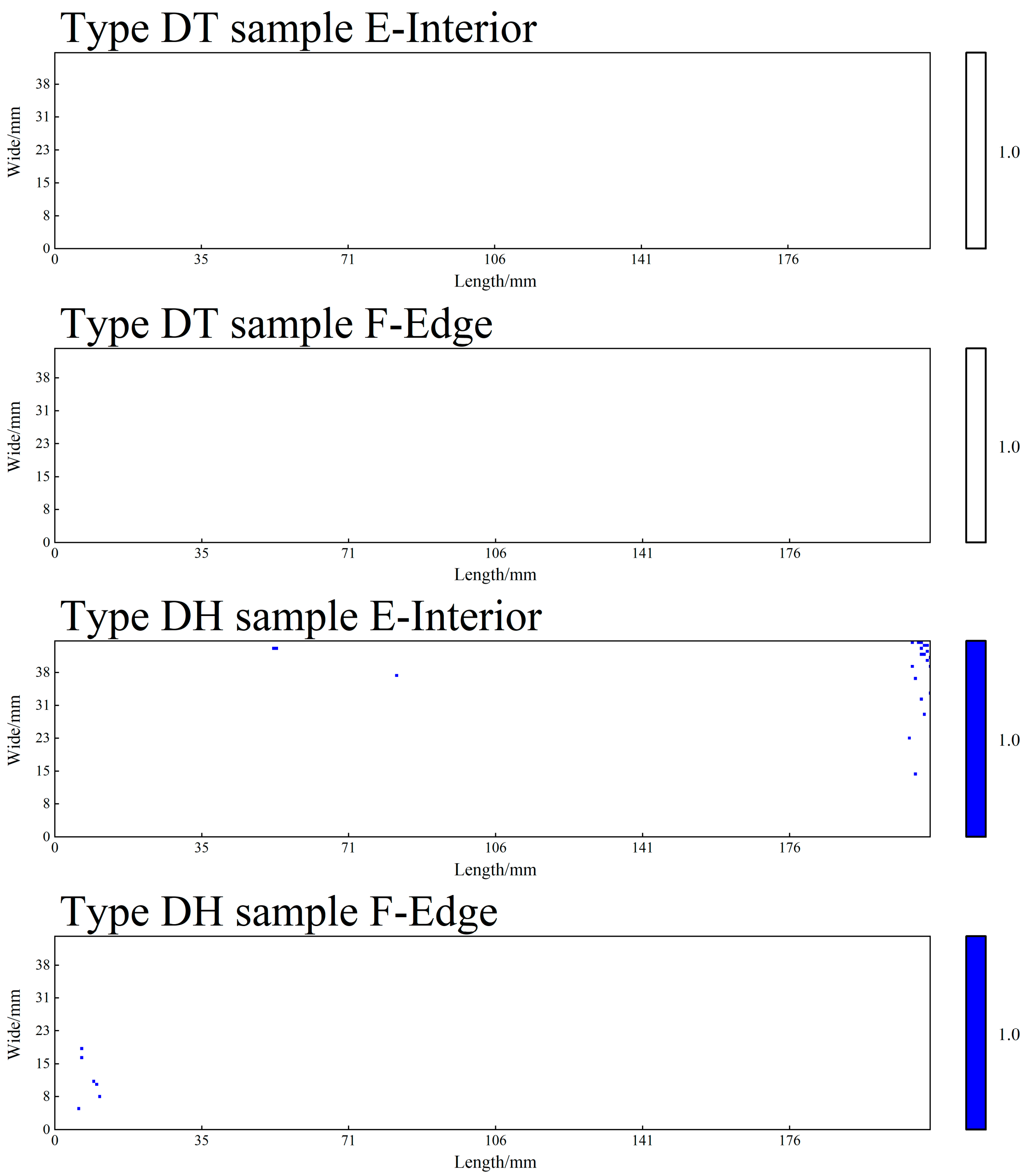

Figure S12. Inclusion rating distribution of Type D inclusions (H and T series) in the dia.810 mm ESR ingot-Head.

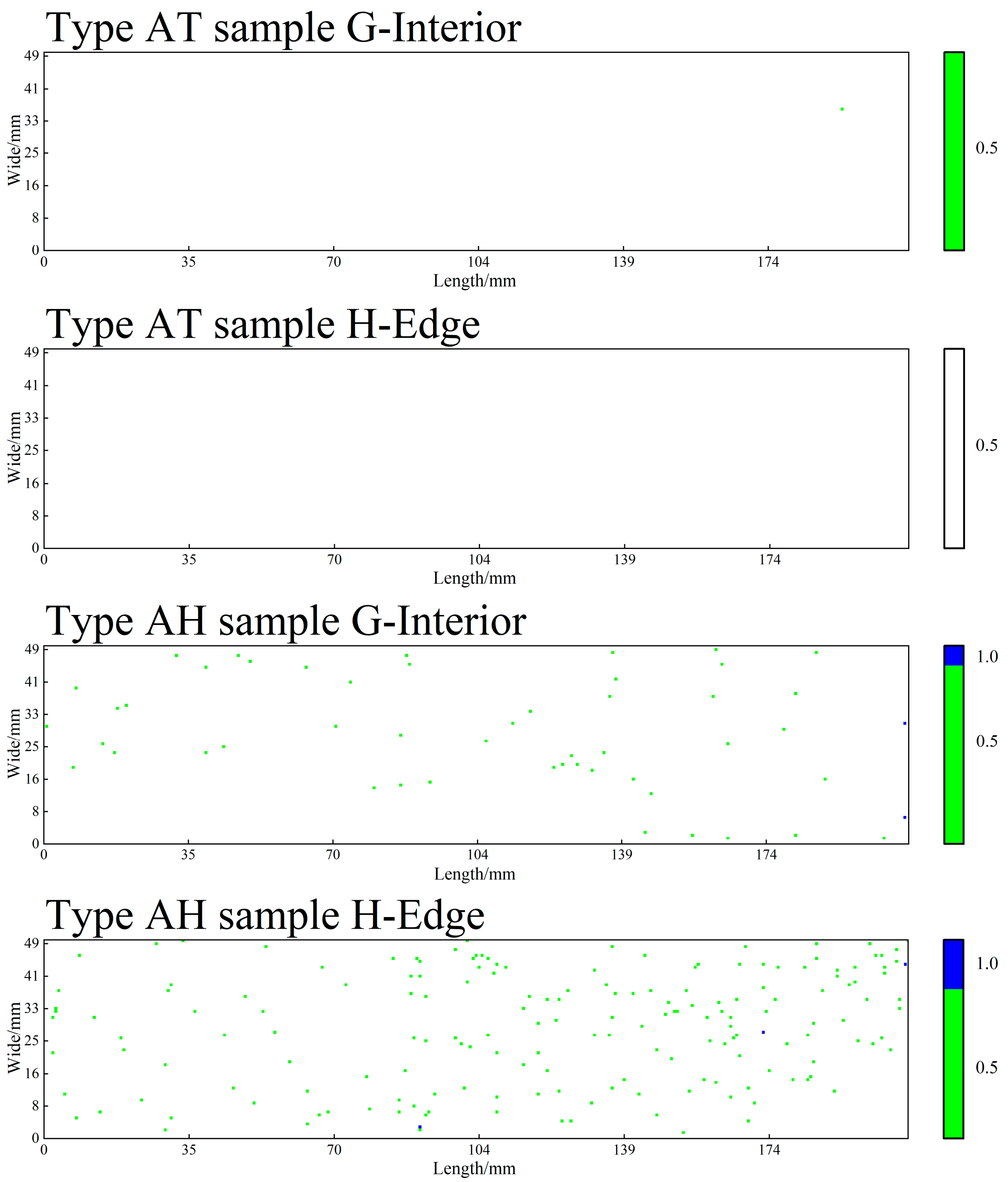

Figure S13. Inclusion rating distribution of Type A inclusions (H and T series) in the dia.810 mm ESR ingot- Tail.

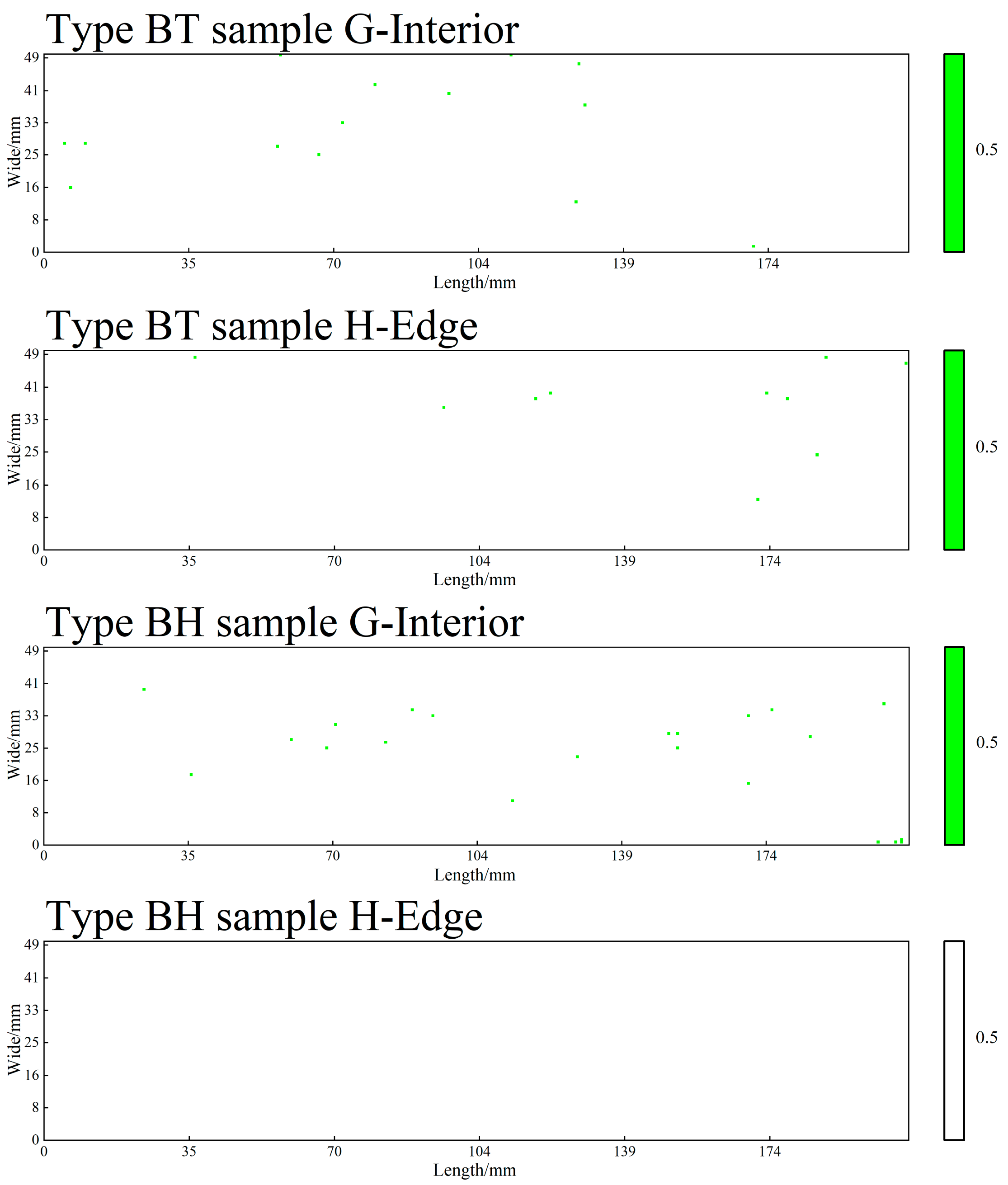

Figure S14. Inclusion rating distribution of Type B inclusions (H and T series) in the dia.810 mm ESR ingot- Tail.

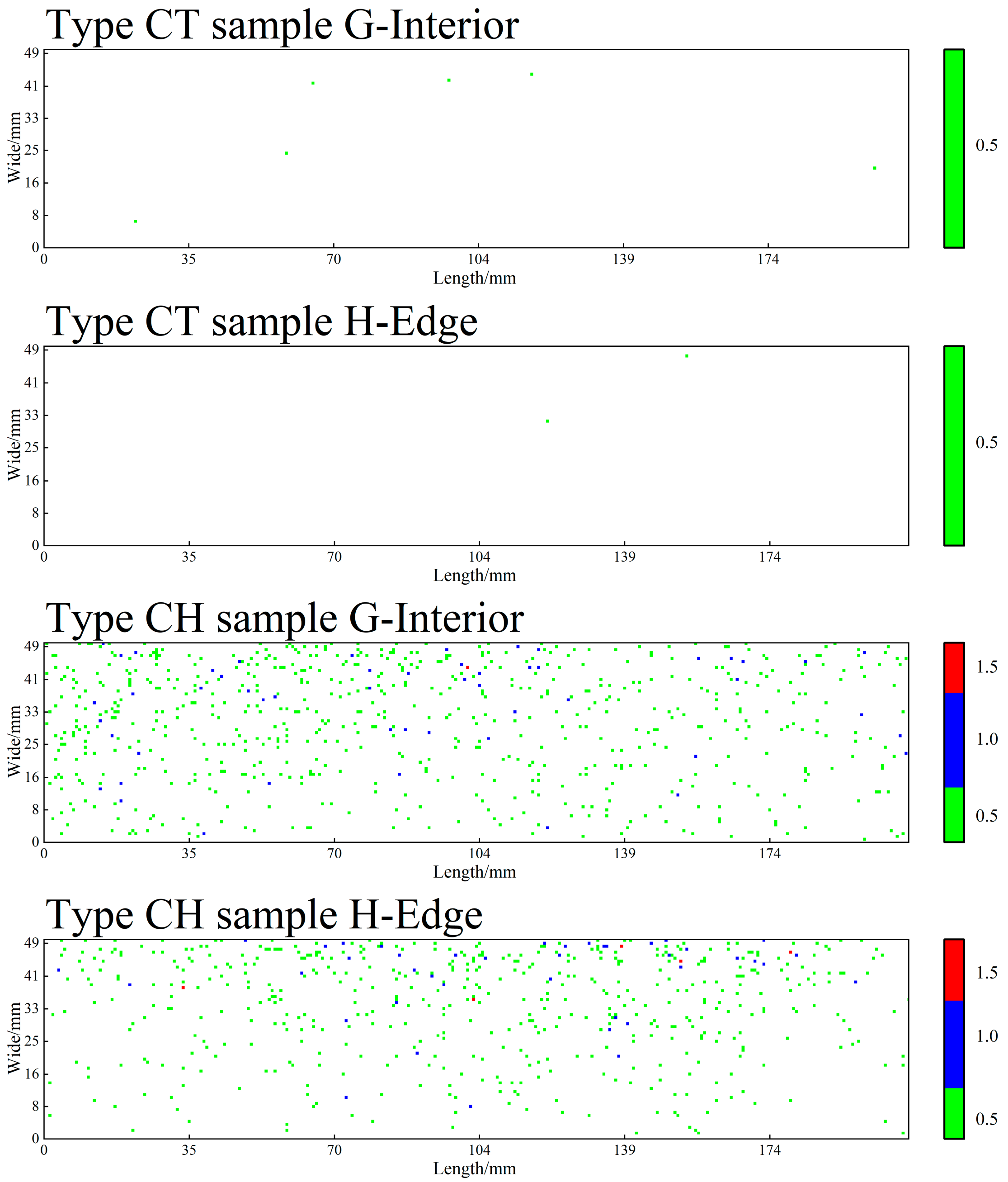

Figure S15. Inclusion rating distribution of Type C inclusions (H and T series) in the dia.810 mm ESR ingot- Tail.

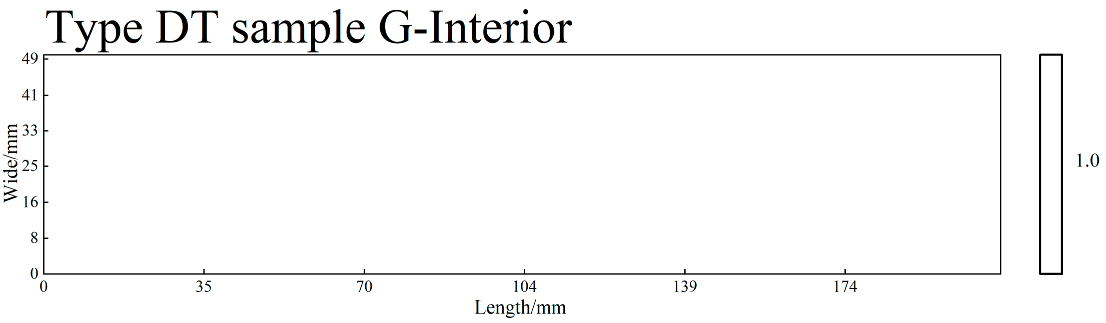

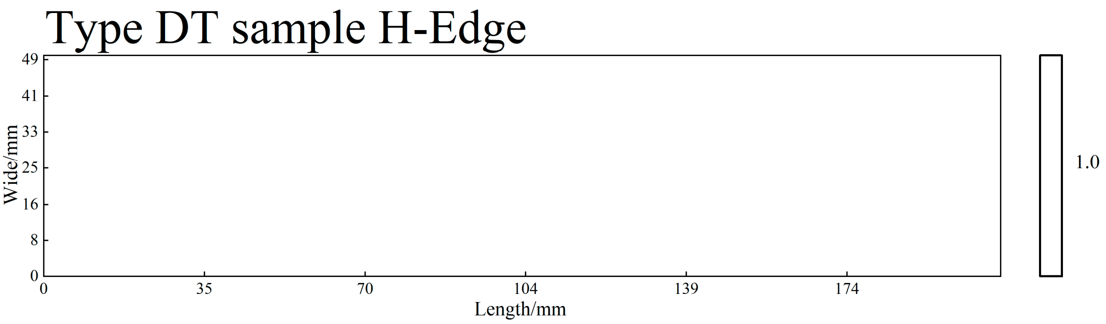

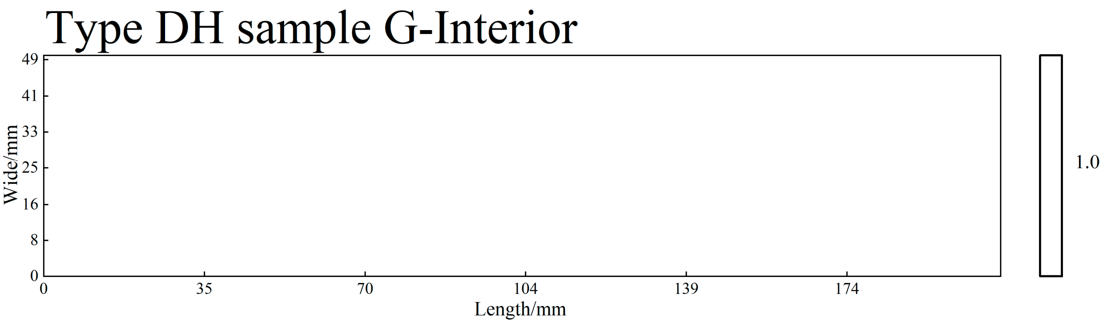

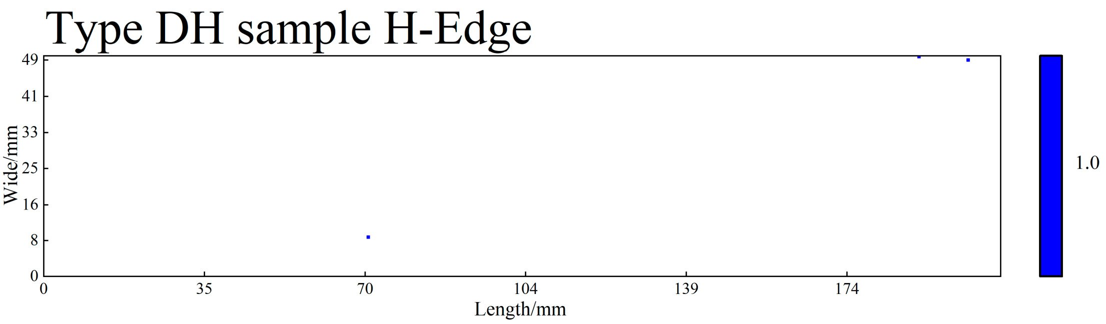

Figure S16. Inclusion rating distribution of Type D inclusions (H and T series) in the dia.810 mm ESR ingot- Tail.

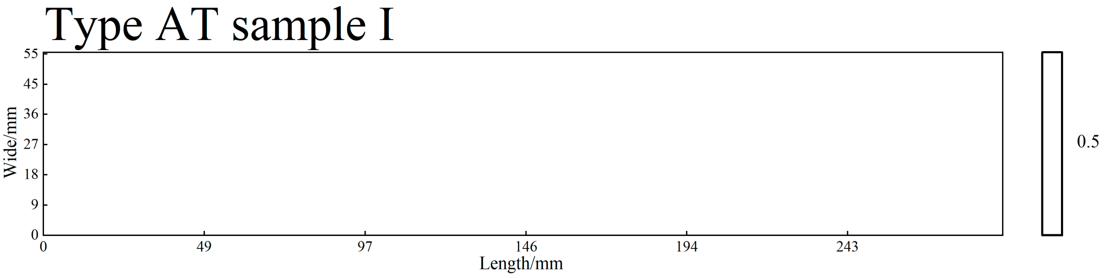

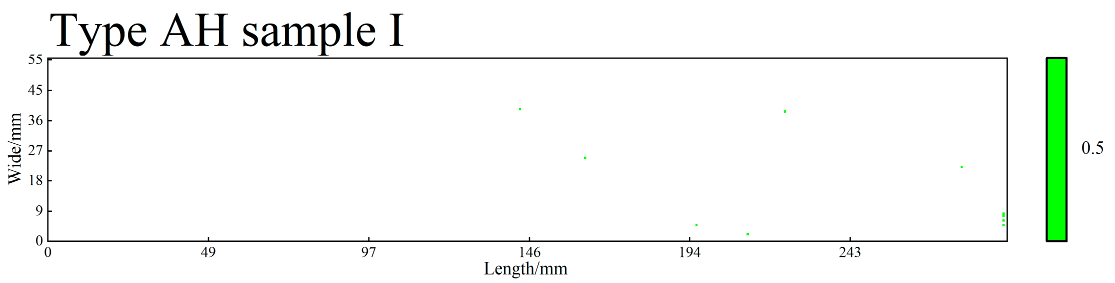

Figure S17. Inclusion rating distribution of Type A inclusions (H and T series) in the dia.400 mm forged billet-Head.

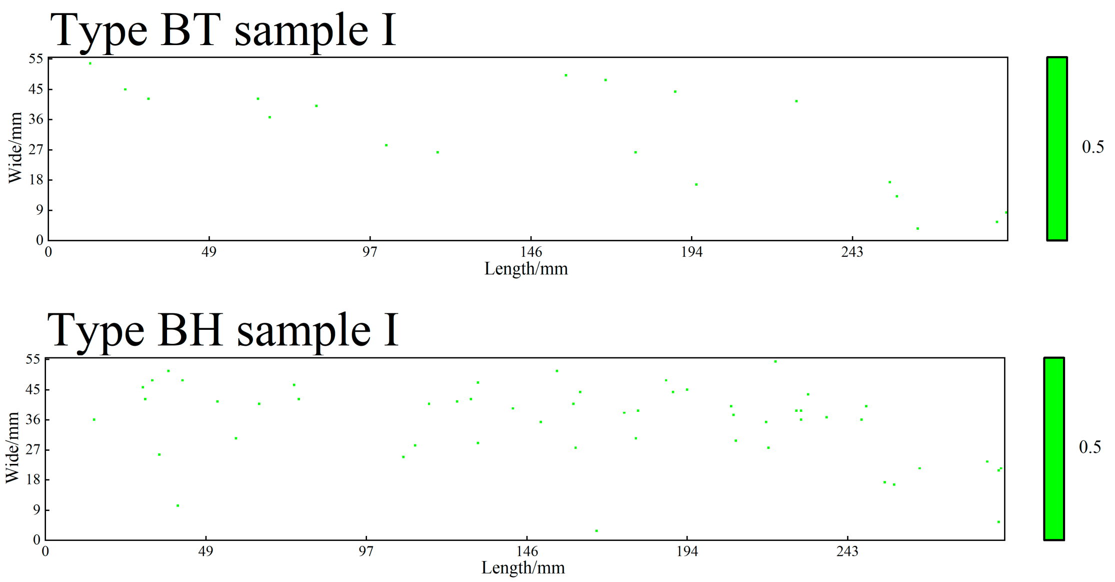

Figure S18. Inclusion rating distribution of Type B inclusions (H and T series) in the dia.400 mm forged billet-Head.

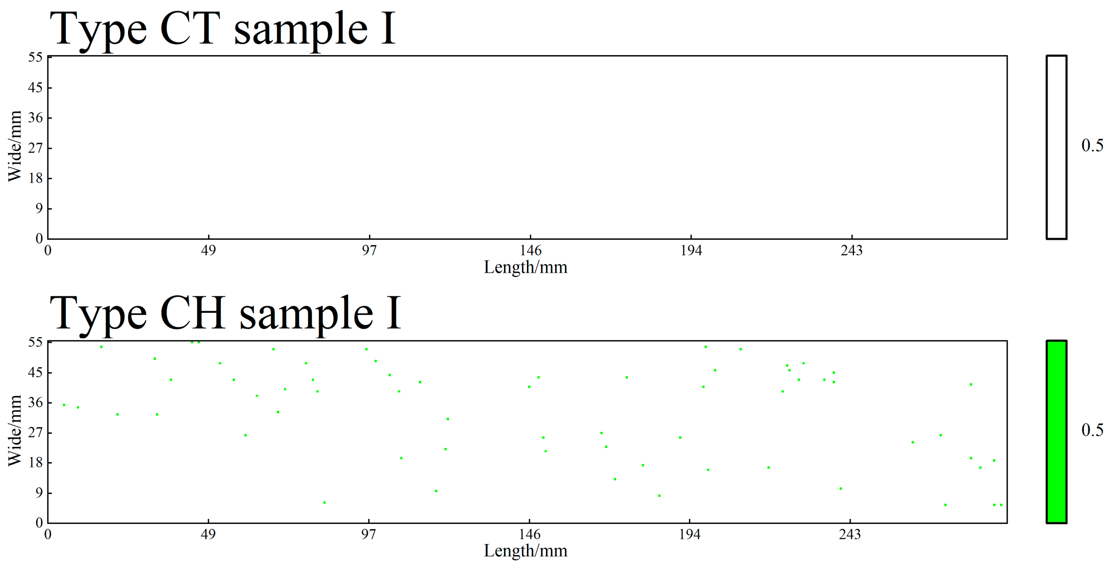

Figure S19. Inclusion rating distribution of Type C inclusions (H and T series) in the dia.400 mm forged billet-Head.

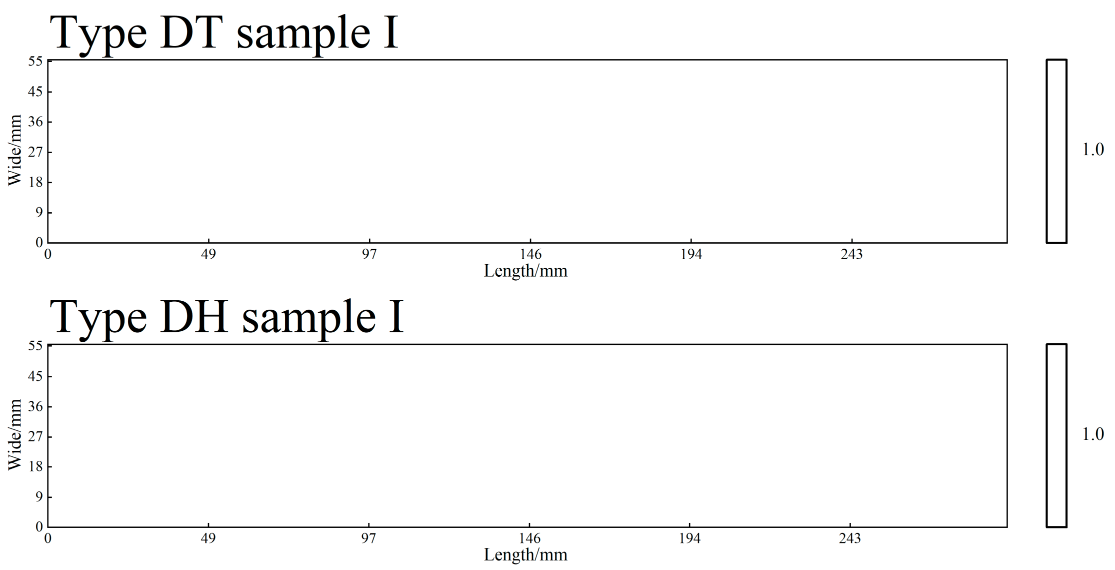

Figure S20. Inclusion rating distribution of Type D inclusions (H and T series) in the dia.400 mm forged billet-Head.

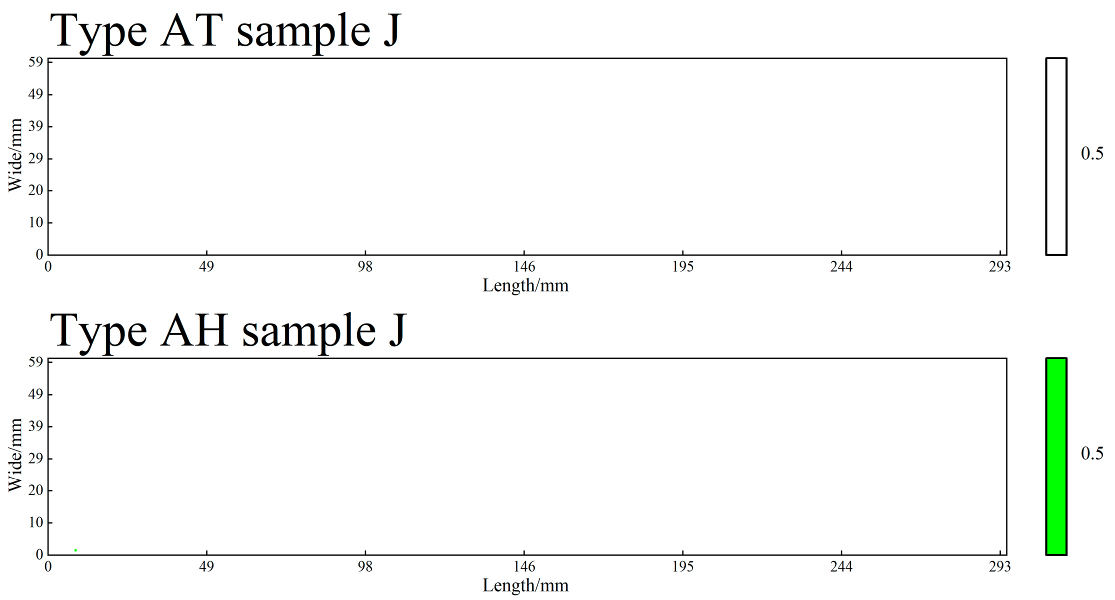

Figure S21. Inclusion rating distribution of Type A inclusions (H and T series) in the dia.400 mm forged billet- Tail.

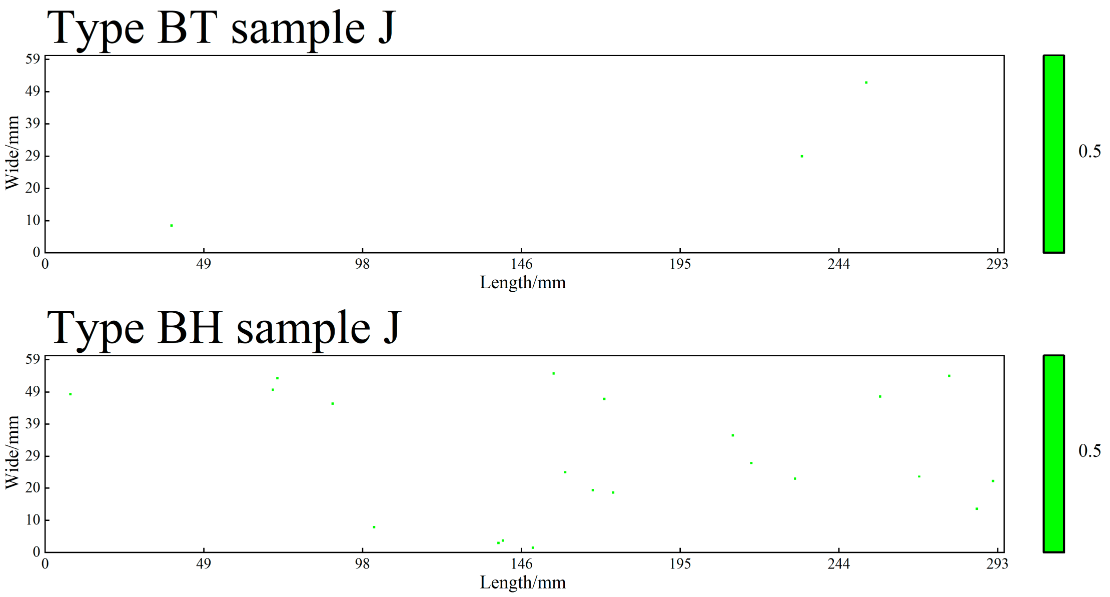

Figure S22. Inclusion rating distribution of Type B inclusions (H and T series) in the dia.400 mm forged billet- Tail.

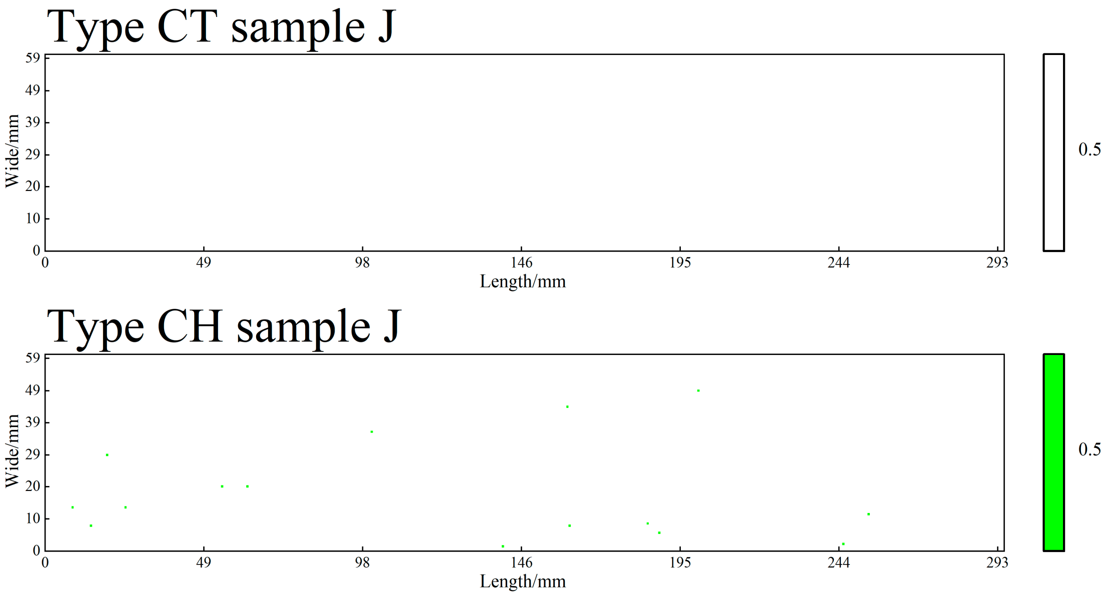

Figure S23. Inclusion rating distribution of Type C inclusions (H and T series) in the dia.400 mm forged billet- Tail.

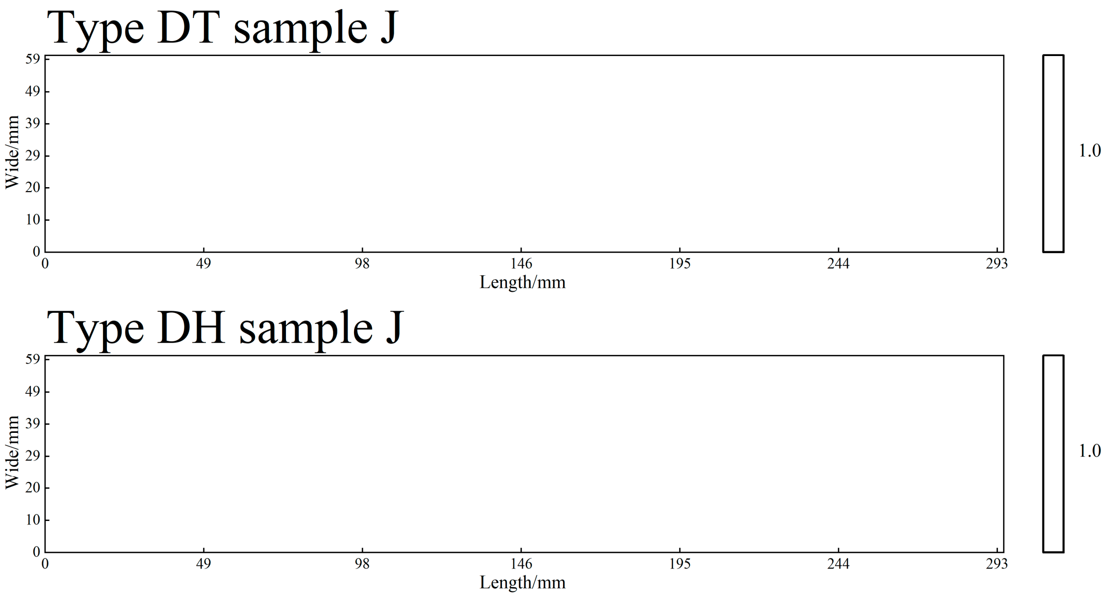

Figure S24. Inclusion rating distribution of Type D inclusions (H and T series) in the dia.400 mm forged billet- Tail.
